# Supplementary material for: RNA synthesis is associated with multiple TBP-chromatin binding events
Source: Sci Rep. 2017 Jan 4;7:39631. doi: 10.1038/srep39631 (PMC5209698; doi:10.1038/srep39631)
Supplement: Supplementary Information [file srep39631-s1.pdf]

Supplementary material for *RNA synthesis is associated with multiple TBP-chromatin binding events*

Hussain A. Zaidi, David T. Auble, Stefan Bekiranov

October 30, 2016

Contents

1 Peak calling, microarray normalization, and scaling **4**

2 Theoretical framework for ChIP-chip competitive binding **4**

2.1 Model of in vivo transcription-factor (TF) induction, competitive binding, and TF-chromatin cross-linking 5

2.2 DNA fragment extraction using ChIP 6

2.3 Hybridization of DNA fragments on a microarray 7

2.4 Optical read-out 7

3 Dependence of Occupancy Ratio on k_a and k_d **8**

4 Ideal solution to competitive in vivo binding **8**

5 Nonlinear fitting algorithm **10**

5.1 Ideal model fitting routine 11

5.2 Bias in ideal solution and numerical look-up table 11

5.3 Numerical Newton's method fitting 12

6 Newton's method exceptions, largest reliable k_d estimates, and competition ChIP residence times **13**

7 Probe-to-gene association algorithm **13**

8 Summary statistics for the nonlinear fitting and filtering procedure **14**

9 Comparison of competition ChIP derived off-rates with Cross-linking Kinetics derived off-rates **15**

10 Transcription rate versus TBP residence time **15**

11 Ribosomal protein genes **16**

12 Comparison of TBP residence times at TATA, SAGA and TFIID genes **16**

13 Comparison with van Werven et al. [1] analysis of TBP turnover **16**

14 Poisson model of turnover **17**

15 Comparison with Rap1 residence times **17**

15.1 Long residence times for Rap1 in Lickwar et al. [4] 18

16 Comparison with histone turnover **20**

17 Presence or absence of transcription factors affecting TBP residence times, transcription rates, and efficiencies **21**

18 Supplementary Figures **23**

1 Peak calling, microarray normalization, and scaling

We analyzed the data set used by van Werven et al. [1] and publicly available under E-M-TAB-58 on ArrayExpress (www.ebi.ac.uk/arrayexpress). The raw data provides the optical signal intensity for induced (HA) and endogenous (Avi) protein concentrations hybridized on a whole-genome microarray. The reported HA induction times are 10, 20, 25, 30, 40, 60, and 90 minutes. For each induction time, the data provides S_{HA} (signal for the induced protein) and S_{Avi} (signal for the endogenous protein) replicated by swapping Cy_3 and Cy_5 dyes to take into account dye-specific variations in the intensity of the optical signal. As a first step to normalizing the data, we subtracted the local background (given as the mean of the background in the optical signal) from S_{HA} and S_{Avi}. Second, we used the geometric average of the ratio S_{HA}/S_{Avi} defined as $R_m = \sqrt{\frac{S_{HA}^{Cy3}}{S_{Avi}^{Cy5}}\frac{S_{HA}^{Cy5}}{S_{Avi}^{Cy3}}}$ to smooth out the significant noise present in individual channel data. The histogram of $\log_2(R_m)$ for the $t = 0$ min data showed a normal-like right edge with a peak at $\log_2(R_m) \sim 0.5$ preceded by a long non-normal tail associated with TBP enriched signal. To identify TBP binding sites (signal probes) we fitted a normal distribution to the identified right edge (background probes) as shown in Supplementary Figure S1 and selected probes in the left tail with a false discovery rate (FDR) of 0.05 or less as the signal probes. We needed to identify non-specific binding background for each value of induction time and normalize the signal data to the background. To accomplish that goal, we fitted a normal to the background probes (selected as described above from the $t = 0$ minute data) for each induction time as shown in Supplementary Figure S1. Dividing the ratio R_m for signal probes by the mean of the normal distribution fitted to the background probes gave us the signal normalized to the non-specific binding (R_m^n). This normalization smoothed the variability of the experimental conditions from one time point to the next, and made the data for all the induction time points comparable for a quantitative dynamical analysis.

In Sec. 2 we motivate that R_m^n is related to the ratio of the in vivo occupancies of the competitor to the endogenous and in 3 we show that R_m^n is effectively independent of k_a for almost all values of k_a (Supplementary Figure S3e–h). Hence, we extract only k_d (i.e., TBP residence time $t_{1/2} = \log(2)/k_d$) from the data we analyzed.

2 Theoretical framework for ChIP-chip competitive binding

The ChIP-chip competitive binding experiment conducted by van Werven et al. [1] can be broadly modeled as comprised of four steps:

- in vivo protein induction, competitive binding, and cross-linking
- DNA fragment extraction using ChIP
- Hybridization of DNA fragments on a microarray

- Optical read-out of DNA concentration on a microarray using flourescent proteins

The following subsections relate the optical signal from microarray channel data to the in vivo occupancies of the induced (HA-tagged) and the endogenous (Avi-tagged) TBP.

2.1 Model of in vivo transcription-factor (TF) induction, competitive binding, and TF-chromatin cross-linking

In competition ChIP [1] competitor TBP is induced over time and competes for binding on genomic loci with the endogenous TBP. The induced species is denoted by a subscript "B" and the endogenous with subscript "A" in the following analysis. The in vivo concentration of induced TBP is a function of time and is denoted by $C^i_B(t)$ (with the superscript "i" denoting "in vivo"), while the concentration of endogenous TBP is a constant (C^i_A). Assuming that the association and dissociation rates of the two species are the same, we could write a mass-action theory of competitive binding as:

$$\frac{d\theta^i_B}{dt} = -k^i_d\theta^i_B + k^i_a C^i_B(t)(1-\theta^i_A-\theta^i_B) \tag{1}$$

$$\frac{d\theta^i_A}{dt} = -k^i_d\theta^i_A + k^i_a C^i_A(1-\theta^i_A-\theta^i_B) \tag{2}$$

where k^i_a is the association rate (on-rate), k^i_d is the dissociation rate (off-rate), θ^i_A is the fraction of available sites occupied by the endogenous species, and θ^i_B is the fraction occupied by the competitor species. Western blotting experiments measuring $C^i_B(t)/C^i_A$ show that the induced concentration ratio is a sigmoid function of time [1], and we modelled it as

$$C^i_B(t)/C^i_A = X(t/t^{ind}_0)^4/(1+(t/t^{ind}_0)^4) \tag{3}$$

Fitting the western blot data to the above model gave $X = 2.233$ and $t^{ind}_0 = 22$ min. We further checked that the fourth power of t in the above sigmoid function was appropriate by fitting sigmoids with first, second, third, fourth, and fifth power of t and determining that the residual was at a minimum with the fourth power. Main text Fig. 3a shows the data and the fitted curve.

In van Werven et al. [1], the proteins are allowed to compete in vivo for (0, 10, 20, 25, 30, 40, 60, 90) minutes (we call them t^i as shorthand for "in vivo time"), after which the cells are cross-linked with formaldehyde for 20 minutes [1]. The effect of formaldehyde induction on competitive binding of proteins in the cell, and hence the effect on competition ChIP signal, is unknown. To test the order of magnitude effect of formaldehyde induction on kinetic parameters, we assumed that formaldehyde halted the production of induced protein. Then a generalization of the CLK model [6] to competitive in vivo binding dynamics and

cross-linking could be described as:

$$\theta_U^i = 1 - \theta_A^i - \theta_B^i - (\theta_A^{xl} + \theta_B^{xl})\Theta(t - t^i) \tag{4}$$

$$\frac{d\theta_B^i}{dt} = -k_d^i\theta_B^i + k_a^i C_B^{i,xl}(t)\theta_U - k_{xl}C_{FH}\theta_B^i\Theta(t - t^i) \tag{5}$$

$$\frac{d\theta_A^i}{dt} = -k_d^i\theta_A^i + k_a^i C_A^i(t)\theta_U - k_{xl}C_{FH}\theta_A^i\Theta(t - t^i) \tag{6}$$

$$\frac{d\theta_A^{xl}}{dt} = k_{xl}C_{FH}\theta_A^i\Theta(t - t^i) \tag{7}$$

$$\frac{d\theta_B^{xl}}{dt} = k_{xl}C_{FH}\theta_B^i\Theta(t - t^i) \tag{8}$$

where $C_B^{i,xl}(t) \sim C_B^i(t)\Theta(t^i - t) + C_B^i(t = t^i)\Theta(t - t^i)$, and $\Theta(t - t^i)$ is a step function (i.e., $\Theta(t - t^i)$ is zero for $t < t^i$ and one for $t \geq t^i$). $C_B^{i,xl}(t)$ represents the model that induction proceeded like a sigmoid function until formaldehyde was added at $t = t^i$, after which induction was instantaneously stopped and $C_B^{i,xl}$ stayed constant at the value $C_B^i(t = t^i)$. The above model including the effects of cross-linking was ill-suited for fitting the data generated by van Werven et al. [1] for the primary reason that the data was not gathered for different cross-linking times, which is required to attempt an estimate of k_{xl} [6]. Hence, we used Eqns. 1 and 2 as the model to fit the data.

To compare the bias in our estimates of k_d because of using the in vivo model (Eqns. 1 and 2) ignoring the additional step of cross-linking, we simulated the ratio θ_B/θ_A by both sets of equations, Eqns. 1-2 and Eqns. 4-8, as shown in Supplementary Figure S3a,b. Supplementary Figure S3c–d show that calling cross-linked ratio as the in vivo ratio could result in an overestimation of k_d by a factor of 3. That said, the kinetic model we have presented can be used to include cross-linking when data with various cross-linking times is generated. It is important to note that the bias is not due to the kinetic theory framework we have chosen. In other words, the Poisson model [7, 4] would also be biased since the effects of cross-linking were ignored when relating the experimental data to the in vivo fractional occupancies of the TBP. By quantifying the effect of cross-linking on estimates of in vivo residence times, we have given a framework to deal with such effects in the future.

Rewriting Eqns. 1 and 2 in terms of C_B^i/C_A^i and redefining k_a^i to $k_a^i C_A$ (such that k_a and k_d both have units of inverse time) we arrived at the equations that were used in all subsequent analyses:

$$\frac{d\theta_B^i}{dt} = -k_d^i\theta_B^i + k_a^i\frac{C_B^i(t)}{C_A^i}(1 - \theta_A^i - \theta_B^i) \tag{9}$$

$$\frac{d\theta_A^i}{dt} = -k_d^i\theta_A^i + k_a^i(1 - \theta_A^i - \theta_B^i) \tag{10}$$

2.2 DNA fragment extraction using ChIP

We assumed that the amount of the fraction of HA-tagged (competitor) and Avi-tagged (endogenous) TBP in ChIP DNA was linearly proportional to the in vivo proportions of the

HA and Avi species. Quantitatively, $\theta_A^C = \beta_A \theta_A^i$ and $\theta_B^C = \beta_B \theta_B^i$, where the superscript C denotes ChIP step, θ_A^C and θ_B^C are the normalized ChIP proportions for Avi and HA species, respectively, and β_A and β_B are the antibody affinities for the two species.

2.3 Hybridization of DNA fragments on a microarray

Competitive hybridization follows the same differential equations as in vivo binding (the values for the kinetic rates and the concentrations of the species are different from the in vivo values). Assuming that the DNA was extracted for HA-tagged TBP and Avi-tagged TBP at a single locus, we once again used kinetic theory to describe microarray competitive hybridization as

$$\frac{d\theta_B^h}{dt^h} = -k_d^h \theta_B^h + k_a^h C_B^h (1 - \theta_A^h - \theta_B^h) \tag{11}$$

$$\frac{d\theta_A^h}{dt^h} = -k_d^h \theta_A^h + k_a^h C_A^h (1 - \theta_A^h - \theta_B^h) \tag{12}$$

where the superscript h signifies the hybridization step in the experiment. C_A^h and C_B^h are proportional to $\theta_A^C(t^i)$ and $\theta_B^C(t^i)$ (again, t^i is the in vivo time at which formaldehyde was added). Since microarray hybridization is carried out over many hours, we assumed that hybridization had reached equilibrium, and found the equilibrium solution by letting t^h approach infinity:

$$\theta_B^h(t^i, t^h \to \infty) = \frac{k_a \beta_B \theta_B^i(t^i)}{k_a(\beta_A \theta_A^i(t^i) + \beta_B \theta_B^i(t^i)) + k_d} \tag{13}$$

$$\theta_A^h(t^i, t^h \to \infty) = \frac{k_a \beta_A \theta_A^i(t^i)}{k_a(\beta_A \theta_A^i(t^i) + \beta_B \theta_B^i(t^i)) + k_d} \tag{14}$$

$$\tag{15}$$

Taking the ratio θ_B^h/θ_A^h, we arrived at

$$\frac{\theta_B^h(t^i, t^h \to \infty)}{\theta_A^h(t^i, t^h \to \infty)} = \gamma \frac{\theta_B^i(t^i)}{\theta_A^i(t^i)} \tag{16}$$

where γ is an undetermined coefficient encoding the ratio of antibody affinities β_B/β_A. Note that Eqn. 16 does not include non-specific binding background, which we account for below.

2.4 Optical read-out

The final step in van Werven et al. [1] is to tag competitor and endogenous species with florescent proteins and optically read out the concentrations of the Avi and HA species by measuring the brightness of the fluorescent proteins at each microarray probe [1]. As described in Sec. 1, each time point is replicated by switching the fluorescent proteins between the HA and Avi species to take into account the binding bias of the dyes. For each

replicate, the mean of the local optical background for each locus was subtracted from the fluorescence signal to take into account inhomogeneities in oligo binding to the microarray. After the optical subtraction, the ratio S_{HA}/S_{Avi} was calculated for each replicate, and the ratios were geometrically averaged over dye-swapped replicates to get the mean ratio (R_m), as discussed in Sec. 1. This ratio R_m was then normalized to the non-specific background in each sample to arrive at R_m^n. We assumed that R_m^n was linearly proportional to θ_B^h/θ_A^h:

$$R_m^n = \alpha\theta_B^h/\theta_A^h + B \tag{17}$$

where α is a linear scale factor (explained in the following) and B is a measure of the background. Substituting in Eqn. 16, we arrived at

$$\alpha R_m^n(t^i) - B = \frac{\theta_B^i(t^i)}{\theta_A^i(t^i)} \tag{18}$$

where we have absorbed γ and redefined α and B. This result is intuitive, and our analysis above shows that it comes out naturally from simple underlying assumptions about the experimental protocol. The value of α at each locus was chosen such that R_m^n approached the asymptotic value of $C_B/C_A = 2.23$ as $t^i \to \infty$. The rationale for this scaling comes from the fact that the in vivo competitive dynamics model predicts the ratio $\theta_B^i/\theta_A^i \to C_B^i/C_A^i$ as $t^i \to \infty$. B was estimated as the y-intercept of a smooth fit to $R_m^n(t)$ (a sigmoid fit, as discussed in Sec. 5). Having related $R_m^n(t)$ to the in vivo occupancy ratio (Eqns. 9 and 10), we could fit the in vivo model to $R_m^n(t)$ as described in the following sections.

3 Dependence of Occupancy Ratio on k_a and k_d

Numerical modeling of Eqns. 9 and 10 showed that for a wide range of k_d values the ratio θ_B^i/θ_A^i was almost insensitive to the value of k_a. Supplementary Figure S3e–h shows that typically the range of k_d where the ratio data was sensitive to k_a was about 0.01/min$<$ $k_d < 0.05$/min. Out of 1173 probes mapped to unique transcription start sites, 92 had k_d between 0.01/min and 0.05/min, and hence, the proportion of mapped probes in the range where k_a could be estimated was less than 8%. For this reason, we can say quite generally that the ratio data R_m^n could only be used to reliably extract k_d. We point out that our theoretical framework does not have any inherent limitations that would inhibit its application to analyzing endogenous and competitor signals individually to get both k_a and k_d if the noise or bias in each channel is managed (e.g, as in high throughput sequencing techniques).

4 Ideal solution to competitive in vivo binding

The differential equations of in vivo competitive binding (Eqns. 9 and 10) could not be solved analytically as $C_B^i(t)/C_A^i$ was a time varying function. While we relied on a numerical non-linear fitting procedure as a last step of the fitting algorithm, we began by finding an

approximate solution (the "ideal" competition ChIP solution) that we used as a starting step in our fitting algorithm. We have dropped superscript i in the following notation, since the only time dynamics we are now considering are the in vivo dynamics (i.e., cross-linking dynamics are ignored). The ideal competition ChIP solution assumed that induction was a step function going from zero to some final constant value C at $t = 0$ minute (i.e., the induction of competitive protein happened instantaneously). Under this assumption, $dC_B/dt = 0$ for all times $t > 0$ minute and the in vivo differential equations could be solved analytically with the boundary conditions $\theta_B(t \to 0) \to 0$ and $\theta_A(t \to 0) = k_a/(k_a + k_d)$. After solving Eqns. 9 and 10 with the ideal competition ChIP assumption, we substituted the full time-varying function $C_B(t)/C_A$ for C at the end. Hence, the ideal solution is not an exact solution of the in vivo differential equations, but captures the first order effect of $C_B(t)/C_A$ on competitive dynamics, and provided a reasonable, but biased, starting point for the full non-linear fitting algorithm.

Defining $z_1 = \exp(k_a + C_{BA}k_a)t$, $z_2 = \exp(k_d t)$, and $C_{BA} = C_B(t)/C_A$ (for brevity) the ideal competition ChIP solution can be written as

$$\begin{aligned}\theta_A(t) =& \frac{k_a}{z_2(1+C_{BA})(k_a+k_d)(k_a+C_{BA}k_a+k_d)}(C_{BA}^2 k_a + z_2(k_a+k_d) \\ &+ C_{BA}(k_a + k_a z_2 + k_d - k_d/z_1 + k_d z_2)) \qquad (19)\\ \theta_B(t) =& \frac{C_{BA}k_a}{z_1 z_2^2(1+C_{BA})(k_a+k_d)(k_a+C_{BA}k_a+k_d)}(-C_{BA}k_d z_2 + (1+C_{BA})z_1 z_2^2(k_a+k_d) \\ &- z_1 z_2(k_a + C_{BA}k_a + k_d)) \qquad (20)\end{aligned}$$

In the limit of $t \to \infty$, C_{BA} goes to a constant equal to 2.23 (Main text Fig. 3a). In the limit $t \to \infty$, the above approximate solution becomes exact and yields $\theta_B^i/\theta_A^i \to C_{BA}$.

The ideal solution predicted that the Taylor series in time of θ_A and θ_B look like:

$$\theta_A \sim \frac{k_a}{k_a+k_d} - \frac{(C_{BA}k_a^2 k_d)t^2}{2(k_a+k_d)} \qquad (21)$$

$$\theta_B \sim \frac{C_{BA}k_a k_d t}{k_a+k_d} - \frac{C_{BA}k_a(C_{BA}k_a k_d + k_d^2)t^2}{2(k_a+k_d)} \qquad (22)$$

While a Taylor series in θ_B/θ_A looks like:

$$\frac{\theta_B}{\theta_A} \sim C_{BA}k_d t - C_{BA}(k_d^2 + C_{BA}k_a k_d)\frac{t^2}{2} \qquad (23)$$

Remembering that $C_{BA} = C_B(t)/C_A \sim t^4$ for small t, we notice that the dependence of θ_A on k_a is O(1), that of θ_B on k_a is O(t^5), and that of θ_B/θ_A on k_a is O(t^{10}). Hence, the ratio of occupancies is predicted to be almost completely independent of k_a for a wide range of k_d values. This gives a mathematical reason for the numerical evidence presented in Sec. 3 for the relative insensitivity of θ_B/θ_A and ratio data $R_m^n(t)$ to k_a.

5 Nonlinear fitting algorithm

The outline of the nonlinear fitting algorithm we devised to analyze competition ChIP data is as follows:

- We subtracted the locus-specific background from the data and scaled the data by a locus-specific scale factor (as described earlier in Secs. 1 and 2). For this scaling and background subtraction procedure, we fitted a sigmoid to each locus given by

$$R_m^n(t) = X_l((t/t_0)^4/((t/t_0)^4 + 1)) + B \tag{24}$$

  where B was the background, X_l the asymptotic value of R_m^n for that locus, and t_0 was the time required for R_m^n to reach half the asymptotic value. In other words, we fitted the same sigmoid-like curve to each locus that we fitted to $C_B(t)/C_A$. We subtracted the background (B) from the locus data and multipled the locus data with a scale factor (α described in Methods and Supplementary Text Sec. 2) such that the asymptotic value of R_m^n was 2.23. As a caveat, plotting $\log(2)/k_d$ versus t_0 showed that there were 53 loci for which k_d estimates did not match the expectation from sigmoid fits (see Supplementary Figure S4c–d). We found the reason to be in excessive background subtraction when fitting the t^4 sigmoid curve. For these loci we used $X_l((t/t_0)^2/((t/t_0)^2 + 1)) + B$ to estimate the scale factor and the background. Notice that no information from this sigmoid fitting is used beyond scaling and background subtraction. The fact that for some loci a different sigmoid curve gave a better fit was to be expected, since we have ignored the effect of cross-linking on the data (i.e., different cross-linking rates k_{xl} could produce data that did not fit a t^4 sigmoid).

- Some loci showed a great amount of noise, hence it was necessary to apply some noise filters to the data. We applied two filters to each locus: 1) we ignored loci for which 50% of the induction time data points had a residual of more than 0.4 when fitted to a sigmoid (a relatively lenient cut-off to remove loci with extreme noise); and 2) we analyzed loci for which the median across induction time points of the relative residual (defined as the magnitude of the difference between the data and the sigmoid fit value divided by the sigmoid fit value) was less than 0.25 (a more stringent cut off applied after nonlinear fitting to select loci for quantitative biological analyses).

- We fitted the ideal solution of in vivo competitive binding (described in Supplementary Text Sec. 4) to the data that passed the lenient residual filter to get an approximate k_d value (called k_d^{app}).

- The ideal solution introduced a systematic bias in the estimated parameters (shown in Supplementary Figure S2a–c). We fixed the bias by using a lookup table that we devised based on a numerical investigation of the bias introduced by the approximate model over a wide range of input k_d and k_a values. The table gave us new "corrected" k_d values we called k_d^{corr}.

- Finally, we fitted the full, non-analytical model by numerically solving the in vivo differential equations and finding the minimum of the sum of squared error (R) between θ_B/θ_A and the scaled data. We devised a numerical version of Newton's method to accomplish the fitting as described in Supplementary Text Sec. 5.3. The starting point for this fitting routine was the k_d^{corr} value. The starting estimate for Newton's method did not provide a good starting point for a few fits, and the method regressed towards the stationary point arbitrarily close to the induction wall at $t_0^{ind} = 22$ min where $R' = dR/dk_d$ as well as $R'' = d^2R/dk_d^2$ go to zero and k_d goes to infinity. For these cases (58 loci) we ran Newton's method with k_d^{app} as a starting point. For 4 of these loci, this starting point failed as well, and we changed the step size to 0.0005 from 0.001, which yielded good fits.

5.1 Ideal model fitting routine

Fitting the ideal solution analytically to the data was problematic unless we provided good starting values to the non-linear fitting routine (NonlinearModelFit in Mathematica [3]). For that, we employed a Taylor series approximation for $\theta_B(t)/\theta_A(t)$ from Eqns. 19 and 20 as a starting point (once again, we have dropped the superscript i as we are modeling in vivo dynamics). We Taylor expanded the expression in small t, which yielded our starting point for the fitting routine: $\theta_B(t)/\theta_A(t) \sim 8.95 \times 10^{-6} k_d t^5 + 4.48 \times 10^{-6} k_d^2 t^6$.

For the regime where C_B/C_A is almost linear in time ($C_B(t)/C_A \sim at$), we used the approximation

$$\frac{\theta_B^i}{\theta_A^i} \sim -\frac{at(-a(-1+\exp(k_d t))k_a t + k_d(1-\exp k_d t(1+at)))}{k_a(1+at)(at+exp(k_d t)) + k_d(at+\exp(k_d t)(a+at))} \tag{25}$$

Where $a = 0.05$ was estimated from the linear rise in sigmoid fit to the Western blot data of $C_B(t)/C_A$ in van Werven et al. [1] (Main Text Fig. 3a). Hence, the procedure for fitting the ideal solution to the normalized, scaled data was:

- We fitted the Taylor series in t to the early time points ($t \leq 25$ minutes)
- Using the estimate for k_d and $k_a \sim 0.1$, we fitted the model with $C_B/C_A \sim at$ (an arbitrary guess for k_a since the curves are theoretically predicted to be almost independent of k_a)
- Using the estimates for k_d and k_a, we fitted the approximate model to the data

5.2 Bias in ideal solution and numerical look-up table

To estimate the bias in fitting the ideal competition ChIP solution to the data, we simulated data by numerically solving the in vivo differential equations (Eqns. 9 and 10), supplying input k_d (k_d^{in}) and the equilibrium chromatin-bound fraction of endogenous species ($\theta_{A_b} = k_a/(k_a + k_d)$) (which determines k_a^{in} for a given k_d^{in}). Normal noise with a standard

deviation of 0.2 was added to each data point and the noisy simulated data was fitted with the ideal induction (approximate) solution using the ideal model fitting routine described in Supplementary Text Sec. 5.1. This process was repeated 200 times. Supplementary Figure S2a–c shows that there was a systematic bias in k_d^{app} estimated from fitting the ideal solution compared to k_d^{in}, while Supplementary Figure S2d–f shows that there was an order of magnitude uncertainty in the output k_a compared to input k_a (as expected theoretically since the ratio θ_B/θ_A is almost independent of k_a, see Supplementary Text Sec. 3).

To fix the bias in k_d from the ideal competition ChIP solution, we used the above simulations to arrive at a look up table. In other words, given an output k_d from ideal induction fit, our look up table gave us a guess as to what the unbiased value of k_d was likely to be. Specifically, we ran the above simulation over an expanded range of k_d^{in} values (that covered the values of k_d^{app} output by the ideal competition ChIP solution fit to the data) and recorded the output biased k_d for a given input k_d. Hence, for a given k_d^{app}, we could guess k_d^{in}. This value of k_d in our table was the corrected k_d, which we called k_d^{corr}. Supplementary Figure S2a–c shows that our correction look up table performed well at fixing the bias as k_d^{corr} was close to the input parameter k_d^{in}.

Simulation of k_a^{out} for various values of k_a^{in} showed that the estimates for k_a^{out} were orders of magnitude off from k_a^{in}, as was expected from the fact that R_m^n was predicted to be almost independent of k_a. Hence, we did not try to extract k_a from the data.

5.3 Numerical Newton's method fitting

Using the above estimate of k_d^{corr}, we implemented a numerical version of 1D Newton's method on k_d to fit the competition ChIP data. One dimensional Newton's method iterates over a regression parameter, say x, with $x_{n+1} = x_n - f(x_n)/f'(x_n)$, where n is the number of iteration and $f(x)$ is the function whose zero is desired, $f'(x) = df(x)/dx$. Our goal was to find the value of k_d that gave the zero of the derivative of the sum of squared residual (R'). For each iteration of the method, we solved the differential equation numerically for $k_d + 0.001$ ("forward" direction) and $k_d - 0.001$ ("reverse" direction), where the starting k_d for the first iteration was k_d^{corr}, and 0.001 was the step size we chose. The step size corresponded to roughly a twentieth of the smallest k_d^{app} we saw in the data. We calculated the residuals R_f and R_r for the forward and the reverse directions, respectively, which allowed us to estimate $R' = (R_f - R_r)/0.002$ along with $R'' = (R_f - 2R + R_r)/(0.001)^2$, where R'' is the double derivative of the residual with respect to k_d. This allowed us to calculate $k_{d_{n+1}} = k_{d_n} - R'/R''$ and iterate over the algorithm. The stopping conditions on the algorithm were $R' \leq 2 \times 10^{-3}$second squared and $k_d > 0$ (to prevent the algorithm from finding negative k_d results) with a maximum of 100 iterations allowed.

6 Newton's method exceptions, largest reliable k_d estimates, and competition ChIP residence times

As mentioned in Sec. 5, the starting estimate for Newton's method , k_d^{corr}, occasionally did not provide a good starting point and the method regressed towards the stationary point arbitrarily close to the induction wall at $t_0^{ind} = 22$ min (that was expected as near the induction wall R' as well as R'' go to zero as k_d goes to infinity). For these cases (58 loci) we ran Newton's method with k_d^{app} as a starting point. For 4 of these loci, this starting point failed as well, and we changed the step size to 0.005 from 0.001, which yielded good fits. Supplementary Figure S4a,c shows k_d against t_0 for the loci before and after correcting the starting point and the step size, respectively. The figure shows that we detected k_d outliers and fixed for this effectively with this strategy. As mentioned above, for 53 loci the background subtraction based on a t^4 sigmoid was too aggressive, which was yielding $t_{1/2}$ values that were significantly smaller than what was expected from t_0 fits to the data. For these loci we used a t^2 sigmoid function for background subtraction and scaling. Supplementary Figure S4c shows the effect of excessive background subtraction yielding $t_{1/2} = \log(2)/k_d$ values significantly smaller than what would be expected from sigmoid fit t_0 values for 53 loci, while Supplementary Figure S4d shows that with proper background subtraction, the $t_{1/2}$ values followed the t_0 values in a non-linear but monotonic fashion.

Supplementary Figure S4a,c also highlights an important aspect of competition ChIP experiments that was discussed in the main text: the residence time resolution of the experiment. The figures show that the largest k_d we could estimate was $k_d \sim 0.53$/minute, which corresponds to a residence time of $t_{1/2} \sim 78$ seconds (and $t_0 \sim 24.5$ minutes). For smaller t_0 (and hence, larger k_d values), we see that estimates for k_d show significant noise. The reason is that for $t_0 \leq 24.5$ minutes, the data starts to follow the induction data (with $t_0^{ind} = 22$ minutes), and Newton's method regressed towards the stationary point close to the induction wall where effectively any value of $k_d \geq 0.5$/minute fitted the data. Hence, the resolution of a competition ChIP experiment is not directly limited by the induction time, which was about 22 minutes in van Werven et al. [1], but by the noise in the induction curve and the data. The resolution can potentially be made sub-minute if the noise is controlled, even with protein induction taking many minutes. The induction time may have an indirect effect on the resolution as errors tend to scale with the size or mean value of data; hence, larger values of induction time may lead to larger effective errors along the t axis (i.e., time after initiation of induction).

7 Probe-to-gene association algorithm

We used the R64-1-1 20110208.gff version of *Saccharomyces cerevisiae* genome annotations from SGD to relate the competition ChIP probes to genes. Probes that overlapped with the $(+350, -50)$ bp region of a gene start site were associated with that gene. If a probe overlapped with multiple start sites, it was associated with the site closest to it. If multiple

probes overlapped with a transcription start site, the probe with the best FDR of the R_m ratio at t=0 minute (Supplementary Text Sec. 1) was associated to the start site.

8 Summary statistics for the nonlinear fitting and filtering procedure

Supplementary Figure S4e–h and Supplementary Figure S5 show representative fits along with IGV plots of $\log_2(R_m)$ showing fast, moderate and slow dynamics. The overall statistics for our analysis are given below.

- total probes = $41,441$
- probes within $(+350, -50)$ bp of gene start sites (promoter probes) = $10,071$
- promoter probes with (FDR <= 0.05)= 7683
- promoter probes associated with a gene, removing duplicate gene overlaps with one probe, and removing duplicate probe overlaps with one gene = 5364
- **Class 1**: genes that passed the TBP noise filters (see Supplementary Text Sec. 5), with t_0 >= 24.5 min. Total number = 1131.
- **Class 2**: genes that passed the noise filters, with t_0 <= 22 min. Total number = 897. These were genes that showed dynamics faster than the induction curve, and we believe an understanding of the effect of cross-linking is required before these loci can be quantitatively analyzed.
- **Class 3**: genes that passed the noise filters, with t_0 >= 22 min and t_0 <= 24.5 min. Total number = 615. These are loci that were "too fast" and only an upper bound of $t_{1/2}$ < 1.3 minute could be achieved. Estimation of $t_{1/2}$ at these loci could be made with less noise in the competition ChIP data and more precise quantification of protein induction with Western blots.

Statistics for Class 1:

- number of Pol3 genes in Class 1= 215. Pol3 genes that divergently overlapped with nearby tRNA genes were excluded due to broad TBP peaks around tRNA genes (see Supplementary Figure S5d), resulting in 205 Pol3 genes for which residence time could be reliably estimated.
- number of Pol2 genes in Class 1= 916
- **Class 1-b**: Pol2 genes in Class 1 that were not divergently overlapping with nearby Pol3 genes. Total number = 794. These were Pol2 loci for which we reliably extracted k_d values.

Table S2: k_d (in min^{-1})

Locus	Poorey et al. [6]	competition ChIP
LOS1	0.1	≥ 0.5
ACT1	0.1	≥ 0.5
URA1	0.02	≥ 0.5
HSC82	0.7	≥ 0.5
SNR6	0.3	0.1

- **Class 1-a**: Pol2 genes in Class 1 not divergently overlapping with nearby Pol3 genes (see Supplementary Figure S5) for which galactose transcription rate [2] was available. Total number = 623. These loci (and subsets where we intersected these loci with other data sets, e.g. as in Supplementary Figure S8) are what we used in our analyses of $t_{1/2}$, transcription rate (TR), and transcription efficiency ($TRt_{1/2}$) throughout the main text and the supplement.

9 Comparison of competition ChIP derived off-rates with Cross-linking Kinetics derived off-rates

Poorey et al. [6] determined TBP off-rates (and residence times) for several loci in yeast by fitting the Cross-linking Kinetics (CLK) model to ChIP-qPCR data over cross-linking time. Supplementary Table S2 summarizes the off-rates that were found in the CLK study along with the off-rates that were determined from our analysis of competition ChIP data.

LOS1, ACT1, URA1, and HSC82 were all too close to the induction curve to reliably estimate k_d using competition ChIP; thus, we give a lower bound on k_d from competition ChIP for these loci. The k_d estimates are qualitatively in agreement with CLK estimates, except for URA1, which showed much slower dynamics in Poorey et al. [6] compared to this study.

10 Transcription rate versus TBP residence time

Out of all the loci in Class 1, Class 2, and Class 3 (see Supplementary Text Sec. 8) transcription rate (molecules of Pol II per minute) data for yeast in galactose [2] was available for 1862 loci. Plotting the transcription rate against t_0 Supplementary Figure S11a shows that the transcription rate may have a non-monotonic relationship with residence time, with a maximum transcription rate occurring for $t_0 \sim 22$ to 24 minutes. Since the rise in transcription rate could be an artifact of a large number of very dynamic loci in the competition ChIP experiment aggregated near $t_0^{ind} = 22$ minutes, this non-monotonic relationship between the transcription rate and residence time (equivalently, response time t_0) can only be confirmed with experiments with a shorter resolution time.

11 Ribosomal protein genes

Out of 135 RP genes annotated for yeast [8], we find 54 in Class 1 (see Supplementary Text Sec. 8), with an additional 61 in Class 2 or Class 3, which suggests that RP genes, like Rap1 genes (see Supplementary Text Sec. 15), were enriched for short TBP residence times of about a minute or less.

12 Comparison of TBP residence times at TATA, SAGA and TFIID genes

In order to make a direct comparison of the effect TATA box sequences, SAGA and TFIID complexes have on estimates of TBP residence times, we analyzed the same SAGA and TFIID subunits, Spt20 and Taf1 respectively, that van Werven et al. [1] used in their study. Out of 623 loci in Class 1-a (see Supplementary Text Sec. 8), we found 45 with SAGA (Spt20) and 578 without SAGA. Similarly, 110 were TATA-containing, and 513 were TATA-less. Of the 623 loci, 522 contained TFIID and 101 did not. TATA-containing loci tended to have a shorter residence time compared to TATA-less loci (Supplementary Figure S8a, KS p-value=0.007). However, the presence of SAGA or TFIID at promoters did not significantly impact TBP residence times (Supplementary Figure S8d,g). TATA-containing promoters had a slightly higher transcription rate (Supplementary Figure S8b; KS p-value = 0.018) and efficiency (Supplementary Figure S8c; KS p-value = 0.166) than TATA-less promoters. Interestingly, while the transcription rate and efficiency were not significantly affected by Spt20 or Taf1 (Supplementary Figure S8e,f,h,i), multiple SAGA and TFIID subunits had a significant impact on one or both transcription rate (Fig. 6d-f) and efficiency (Fig. 6g-i).

13 Comparison with van Werven et al. [1] analysis of TBP turnover

We found 183 loci with reliable k_d estimates in our analysis where TBP turnover estimates were made by van Werven et al. [1]. Our k_d estimates correlated well with the turnover parameter r of van Werven et al. [1] (Pearson coefficient: 0.46, Kendall tau coefficient: 0.35, Spearman coefficient: 0.52). Supplementary Figure S6a shows k_d versus r and highlights the correlation between the two sets of values. However, there are substantial differences in our modeling, normalization, and scaling approach compared to van Werven et al. [1]. The turnover parameter r is influenced significantly by the value of the ratio (HA/Avi) at $t = 0$ minutes and $t = 10$ minutes. This correlation is negative for Pol II genes and positive for Pol III genes as shown in Supplementary Figure S6b–d. Our analysis does not show any correlation between the signal at $t = 0$ minute and k_d, as shown in Supplementary Figure S6e–g. r is correlated with nascent transcription rate (TR) [2], however, given that the turnover for Pol II genes was strongly negatively correlated with the $t = 0$ min data, any correlations could be due to occupancy levels rather than dynamics.

14 Poisson model of turnover

The Poisson model of turnover with turnover parameter λ (units of 1/minutes) has been applied to competition ChIP data in multiple studies [4, 7]. Monte Carlo simulations of competition ChIP at a given binding site followed by fitting the Poisson turnover model reveal that λ^{-1} is strongly correlated with, and approximates, the physical residence time [4]. Below we show using formal reaction rate theory (this is how k_a and k_d are formally defined) that λ is equal to k_d plus a time dependent factor which yields a modestly biased estimate of $t_{1/2}$ when using λ^{-1} to approximate $t_{1/2}$.

Define $P(t) = \theta_b/(\theta_a + \theta_b)$, which can be interpreted as the fraction of bound sites occupied by the competitive transcription factor. If we define $\theta_{occ} = \theta_a + \theta_b$ as the fraction of sites occupied, and $\theta_u = 1 - \theta_{occ}$ as the fraction of site unoccupied by TBP, then using Supplementary Text Eqs. 1 and 2 one could show that

$$\frac{dP(t)}{dt} = k_a(C_A + C_B(t))\frac{\theta_u}{\theta_{occ}}\left(\frac{C_B}{C_A + C_B} - P(t)\right) \tag{26}$$

Using Eqs. 1 and 2 one can also show that

$$\frac{d\theta_{occ}}{dt} = k_a(C_A + C_B(t))\theta_u - k_d\theta_{occ} \tag{27}$$

Combining the above two equations, we arrived at:

$$\frac{dP(t)}{dt} = \left(k_d + \frac{1}{\theta_{occ}}\frac{d\theta_{occ}}{dt}\right)\left(\frac{C_B}{C_A + C_B} - P(t)\right) \tag{28}$$

Comparing the equation above with Dion et al. [7], we see that $\lambda(t) = k_d + \frac{1}{\theta_{occ}}\frac{d\theta_{occ}}{dt}$, i.e., k_d plus the rate of change of the total occupancy of A and B divided by the total occupancy for any given site. Our numerical simulations showed that the Poisson model is asymptotically exactly equivalent to the kinetic theory (Supplementary Text Eqns. 1 and 2) for very high, potentially unphysical, occupancies. In the range of k_d values that we derive from competition ChIP data, the Poisson turnover model introduces an error that is, at worst, a factor of 2 of the input k_d as shown in Supplementary Figure S9e–h. In other experiments where k_d could be expected to be less than the range we are seeing, the error may be more significant.

15 Comparison with Rap1 residence times

Out of 439 binding sites analyzed in Lickwar et al. [4], 191 Rap1 binding sites overlapped with TBP loci in Class 1, Class 2 or Class 3. 76 sites were seen in Class 1-b loci. Supplementary Figure S11b shows Rap1 residence times [4] against the t_0. We see that most of the loci with Rap1 are concentrated near the fast edge of TBP turnover. This could be an indication that loci with Rap1 have a characteristic TBP residence time that is towards the short time scale of about a minute or less.

Comparing the 76 loci in Class 1-b with Rap1 against those where Rap1 was not detected, we saw that even though TBP residence time was mildly different between the two sets, the transcription rate and the transcription efficiency were markedly different, as shown in Supplementary Figure S8m–o.

15.1 Long residence times for Rap1 in Lickwar et al. [4]

Even though the correlation between the TBP residence time ($t_{1/2}$) and Rap1 relative residence time (λ^{-1}) reported in Lickwar et al. [4] was significant, we saw that TBP residence times $t_{1/2} = (\ln(2)/k_d)$ were on a scale of a few minutes in contrast to Rap1 relative residence times which are on the scale of tens of minutes (throughout, by 'Rap1 relative residence times' we mean λ^{-1}). Given that Rap1 relative residence times appear to be an order of magnitude longer than TBP residence times, we decided to investigate this discrepancy. We found that the cause of the discrepancy could be traced back to the step of scaling the background subtracted and normalized ChIP values in Lickwar et al. [4] to arrive at fractional occupancies which appear in the Poisson turnover model. More specifically, the fractional occupancies used in the theoretical Poisson turnover model satisfy specific criteria at long (approaching infinite) times after induction of the competitor which the normalized ChIP value in Lickwar et al. [4] do not seem to satisfy. We describe this argument in detail in this section.

The central equation used in modeling Rap1 turnover data is Eqn. 9 in Lickwar et al. [4], which is reproduced here for convenience:

$$\frac{d}{dt}P(t) = \lambda \left(\frac{A(t)}{A(t) + B(t)} - P(t) \right) \tag{29}$$

where $P(t)$ is the probability that a competitor occupies a given locus (which is equivalent to the fraction occupied by the competitor divided by the total occupied fraction by the competitor and the endogenous species), $A(t)$ is the concentration of the Myc (competitor) Rap1, and $B(t)$ is the concentration of the Flag (endogenous) Rap1. As an aside, and for the sake of clarity, note that this convention is reverse of the convention we have used for our analysis of TBP residence times where A and B denote endogenous and competitor proteins, respectively. $P(t)$ is related to the ratio of the competitor over the endogenous bound protein ($R(t)$) by $P(t) = R(t)/(1 + R(t))$. $R(t)$ is in turn related to the measured ChIP fraction $mR(t)$ of competitor/endogenous by Eqn. 6 in Lickwar et al. [4]:

$$mR(t) = \frac{R(t) + C_0(1 + R(t))}{1 + C_0(1 + R(t))} \tag{30}$$

where $C_0 = mR(0)/(1 - mR(0))$. Eqn. 30 implicitly assumes that the antibody affinity for the Myc and Flag species is the same, and hence, a global scale factor is implicitly set to 1. We believe that this is a strong assumption that is likely incorrect for the data generated in Lickwar et al. [4]. We highlight that by using the data from RPS29B promoter presented in

Fig. 2e in Lickwar et al. [4]. In the following, we first concretely explain the need for scaling the data using RPS29B as an example. To show the effect of scaling on residence times, we analyzed the data for RPS29B in Lickwar et al. [4] two ways: first, by applying our model to the unscaled data, and second, by applying our model to scaled RPS29B data. These two analyses showed explicitly that the residence time was affected significantly by scaling, and in general, the residence time obtained from scaled data was shorter than that from unscaled data, explaining the discrepancy between TBP and Rap1 residence times. Finally, we showed that our kinetic model applied to Lickwar et al. simulated data [4] gave residence times in agreement with Lickwar et al. [4] simulation input residence time of 1000s. Since the simulated data in Lickwar et al. [4] does not have to be scaled (owing to the lack of antibody effects in simulated data) the agreement between the residence times showed that the difference in the scale of TBP residence times and Rap1 relative residence times did not arise from differences in theoretical models, and the underlying reason for the discrepancy was the scaling factor set to one.

To show that experimental data generated in Rap1 competition ChIP experiments in Lickwar et al. [4] needed to be scaled, we looked at the $t \to \infty$ limit of the Poisson turnover model, i.e., Eqn. 29. In the limit $t \to \infty$, $dP(t)/dt \to 0$, and Eqn. 29 yields $P(t \to \infty) = A(t)/(A(t) + B(t))$. Hence, $R(t \to \infty) = \lim_{t\to\infty} A(t)/B(t)$. Fitting a Hill sigmoid with $n = 4$ to the Myc/Flag protein concentration ratio presented in Fig. 1d of Lickwar et al. [4] yielded a saturation value for the ratio of 2.6. Supplementary Figure S10a shows the sum of squared residuals for the sigmoid fit, from which we ascertained that the saturation value of 2.6 was indeed a minimum of the residuals. Using the RPS29B promoter data presented in Fig. 2e of Lickwar et al. [4], we estimated $C_0 \sim 0.2$ (the average of the first three time points, used as an estimation of the background). Using Eqn. 30, we saw that the asymptotic value predicted by the Poisson model with $R(t \to \infty) \to 2.6$ for $mR(t)$ was approximately 1.93.

On the other hand, when we fitted a sigmoid directly to the $mR(t)$ ratio data at RPS29B promoter presented in Fig. 2e of Lickwar et al. [4], we found that the saturation for $mR(t)$ based on the normalized ChIP ratio data was 0.6 as shown in Supplementary Figure S10b. We note that this long time value of $mR(t)$ is markedly different from the saturation value of 1.93 which was based on our Hill sigmoid fit to the concentration ratio data (discussed above). Thus, calculating the long time (asymptotic) ChIP ratio value for $mR(t)$ using the normalized ChIP ratio and concentration ratio data, separately, yielded discordant values for $mR(t \to \infty)$ of 0.6 and 1.93, respectively. This suggested a disconnect between the asymptotic behavior of the normalized ChIP ratio data and the Poisson model used to fit that data.

Next, we took the data from Lickwar et al. [4] Fig. 2e and fitted our kinetic model to the data two ways. In one fit, we scaled the data so that $mR(t)$ at $RPS29B$ promoter saturated at 1.93, while in the alternative method, we fitted the unscaled $mR(t)$ as presented in Fig. 2e of Lickwar et al. [4]. Supplementary Figure S10c shows that with scaling the data lead to $1/k_d$ that was too short to be measured accurately, probably in the range of one to two minutes. This was due to the scaled ChIP ratio curve rising and saturating as fast or faster

than the concentration ratio of induced over competitor Rap1. On the other hand, our fitted value of $1/k_d \sim 200$ min to the unscaled data is in line with the (λ^{-1}) of 99 min in Lickwar et al. [4].

Finally, as mentioned earlier, we fitted our kinetic model to the simulated data in Lickwar et al. [4] Fig. S.6f with an input λ^{-1} of 1000s. Since the simulated data in Lickwar et al. [4] naturally scales to the appropriate asymptotic value, we expected our kinetic fit to Lickwar et al. [4] Monte Carlo simulated data to produce $1/k_d$ values comparable to Lickwar et al. [4] λ^{-1}. Supplementary Figure S10f shows the kinetic model fit which gave $1/k_d = 882s$, in line with the input λ^{-1} of $1000s$. Supplementary Figure S10g also shows that using $1000s$ for $1/k_d$ produced essentially the same fit within noise in the data, and hence, our estimate of $1/k_d$ was in agreement with the input parameters to the simulation.

In summary, our analysis showed that scaling the experimental $mR(t)$ so that $P(t) \rightarrow A(t)/(A(t) + B(t))$ as $t \rightarrow \infty$ is an important step in analyzing competition ChIP data. If we look at Fig. 2e in Lickwar et al. [4], we can now understand why the fit to the unscaled data at RPS29B is almost linear with no saturation in sight: the theory expects the saturation to be much higher than 0.6, and hence, produces a fit that treats the non-linearity in the data between $t = 30$ min to $t = 120$ min as noise. Since the scaling factor comes from antibody affinity and other differences for the Myc and Flag species, which should be comparable regardless of the genomic locus probed, the lack of scaling in Lickwar et al. [4] does not acutely affect the global correlation that we see between TBP $t_{1/2}$ and Rap1 λ^{-1} (though moderate effects on correlation are possible), but does introduce a bias factor of 20-30 times for Rap1 relative residence times, λ^{-1}, [4] compared to actual Rap1 residence times, $t_{1/2}^{Rap1}$. A caveat to this analysis is that we estimated the saturation of the ChIP ratio by fitting a sigmoid to the RPS29B data that may not have saturated in the time scale shown. Hence, it is possible that if data is taken past 150 min. in Lickwar et al. [4], the RPS29B data shows saturation near the saturation of the protein concentration ratios. Similarly, the protein induction curve in Fig. 1d in Lickwar et al. [4] does not show conclusive signs of saturation, and our estimate of the saturation value for the protein concentration ratios may be imprecise.

16 Comparison with histone turnover

Dion et al. [7] reported 794 loci where +1 nucleosome histone turnover was measured. We found 377 in our data in Class 1 (see Sec. 8). Of these 377 loci, transcription rate data for yeast grown in galactose [2] was available for 307 loci (i.e., 307 loci in Class 1-a), which were used in the +1 nucleosome analysis in the main text. When looking at all the TBP sites that passed the noise filters regardless of t_0 (Class 1, Class 2, or Class 3 - see Sec. 8), we saw 1220 sites where +1 nucleosome turnover was available. Supplementary Figure S11c shows the +1 nucleosome turnover against t_0 for these sites, where we see that there may be a non-monotonic relationship between +1 nucleosome turnover and TBP turnover.

17 Presence or absence of transcription factors affecting TBP residence times, transcription rates, and efficiencies

The genome-wide binding map of 202 transcription factors (TFs) provided in Venters et al. [5] was used to assess the effect of transcription factors on TBP residence times, transcription rates, and transcription efficiencies. We found 62 that lead to high (fast) and 8 that lead to low (slow) TR or $TRt_{1/2}$. Total number of orchestration, initiation, access, and elongation factors among the 202 total TFs was 41, 43, 83, and 34, respectively.

While we found only one TF (Tfa2) out of 202 whose presence or absence at the promoter of genes yielded significantly different TBP residence times (Supplementary Figure S12a), we found $\sim 50\%$ of all the mapped initiation and elongation factors significantly modulated transcription rate and efficiency. These included subunits of the PIC assembly complexes TFIID, TFIIF, TFIIH and Pol II, while enrichment of TFIIE at promoters displayed increased transcription rate only (Supplementary Figure S12e,f). In addition, presence of multiple factors in complexes associated with elongation as well as initiation at promoters including Spt6, FACT and Mediator showed higher transcription efficiency and rate (Supplementary Figure S12e,f).

Components of complexes in the "access" class–those that regulate histones and chromatin [5]–associated with establishing active states of chromatin including monoubiquitination of H2BK123 (Bre1 and Htb1), trimethylation of H3K4 (COMPASS), acetylated histone tails (SAGA, NuA4 and Ada) and chromatin remodeling (Isw1 and Nhp6a) were enriched at genes with higher transcription efficiency and rate (Supplementary Figure S12e,f). In contrast, we also found access factors associated with chromatin and transcriptional silencing including a histone deacetylase (Sir2), and proteins that directly interact with histones (Tup1 and Spt2) at genes with higher transcription rates and efficiencies (Supplementary Figure S12e). Moreover, we found the presence of the histone variant Htz1 and components of the SWR1 complex, which exchanges H2A.Z (Htz1) for H2A in chromatin, at promoters were associated with lower transcription rates and efficiencies (Supplementary Figure S12e,f). While this result is consistent with genomic studies that show an inverse correlation between Htz1 and gene expression levels [9], deletion of Htz1 reduces the rate of Pol II elongation 24% at the GAL10p-VPS13 gene [10]. Finally, in the "orchestration" class–sequence specific activators and repressors [5]–we found that the presence of factors (Sfp1, Ifh1 and Rap1) that are associated with activating ribosomal protein and biosynthesis genes resulted in higher transcription rates and efficiencies while a factor that activates phosphatase metabolism (Pho2) only increased transcription rates (Supplementary Figure S12e). Interestingly, factors that recruit the repressive Tup1-Cyc8 complex (Cin5 and Skn7) along with Tup1 were associated either only with increased transcription rate or both increased transcription rate and efficiency (Supplementary Figure S12e). We note that an important caveat in this analysis, which may explain the enrichment of a few transcriptional repressors at genes with higher transcription rates and efficiencies, was that the transcription rates and efficiencies were calculated for yeast in galactose [1, 2] while the 202 TFs were mapped in yeast in YPD [5]. While there

are likely many genes whose TBP, PIC and transcription dynamics are not altered by the differences in media, there are some whose regulatory factor and transcription dynamics are significantly altered. This may explain the presence of a small number of repressors (Sir2, Tup1, and Spt2) from both the "access" and "orchestration" class significantly increasing transcription rate and efficiency in our study. Indeed, transcription rates and efficiencies might increase for yeast in galactose compared to YPD at genes where these repressors are absent at their promoters in galactose and present at their promoters in YPD. Finally, we note that the significance associated with the few TFs that uniquely modulated transcription efficiency was notably less than that of the TFs that modulated transcrption rate (Supplementary Figure S12d). This together with the fact that the majority of the signifcant TFs shown in Supplementary Figure S12d-e modulate both transcription rate and efficieny suggests that presence or absence of individual TFs affects transcription rate and, to a much lesser extent, transcription efficiency.

18 Supplementary Figures

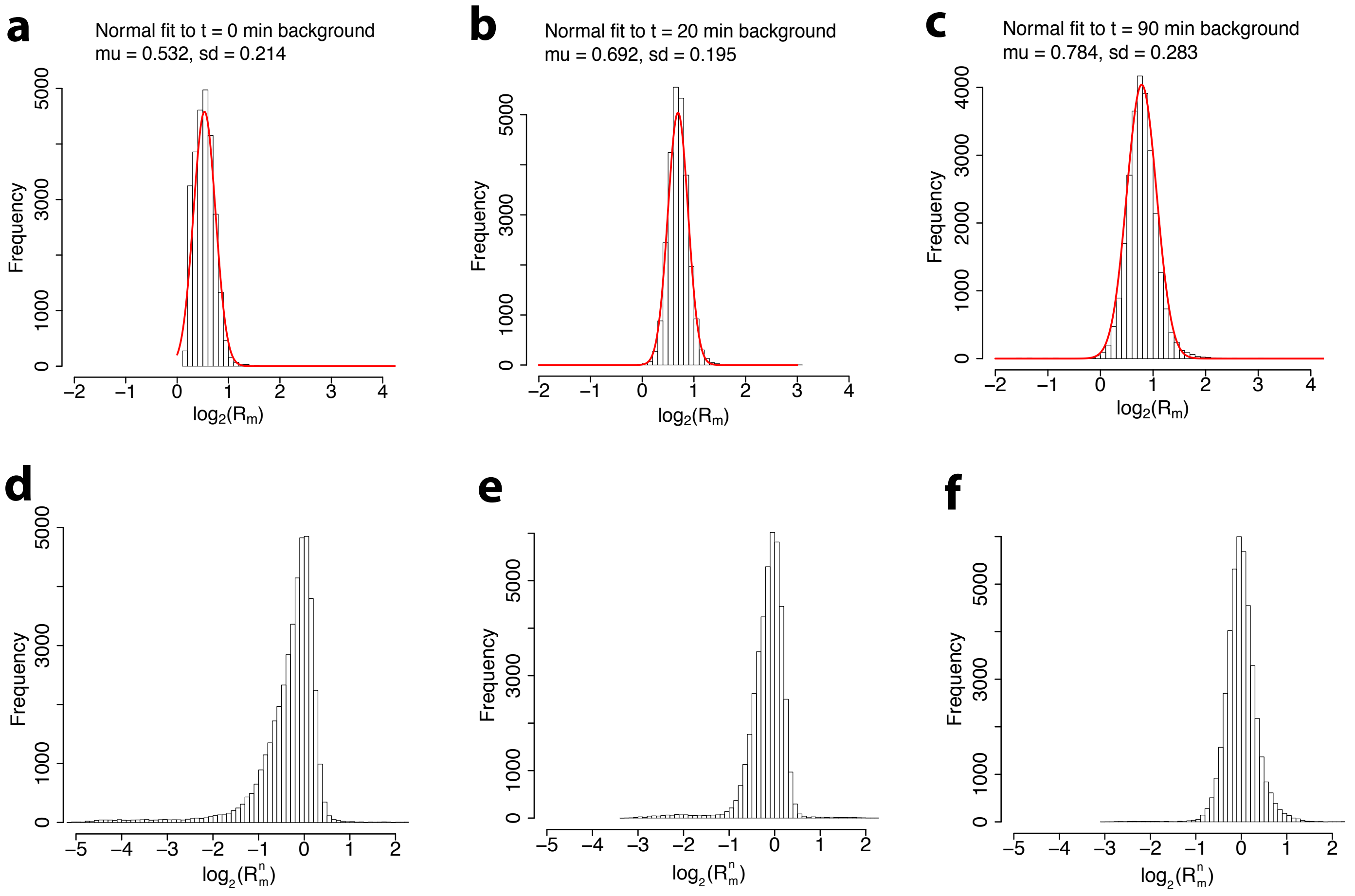

Supplementary Figure S1: Background estimation and normalized data histograms for induction times of 0, 20 and 90 minutes. The data shown was geometrically averaged to smooth out channel-specific noise in the competition ChIP experiment [1]. **a − c,** $\log_2(R_m)$ non-specific background probes chosen from the $t = 0$ minute data fit the normal distribution well for all time points. The mean of the normal distribution was used to normalize the data for each time point. **a,** Background probes were selected by fitting a normal distribution to the right edge of the $t = 0$ data histogram. These probes were used as background probes for all time points. **b,** Fit to background probes at t = 20 min. **c,** Fit to background probes at t = 90 min. **d − f,** $t = 0$ min, $t = 20$ min, and $t = 90$ min data after normalization showing the mode of the distributions at $\log_2(R^n_m) = 0$.

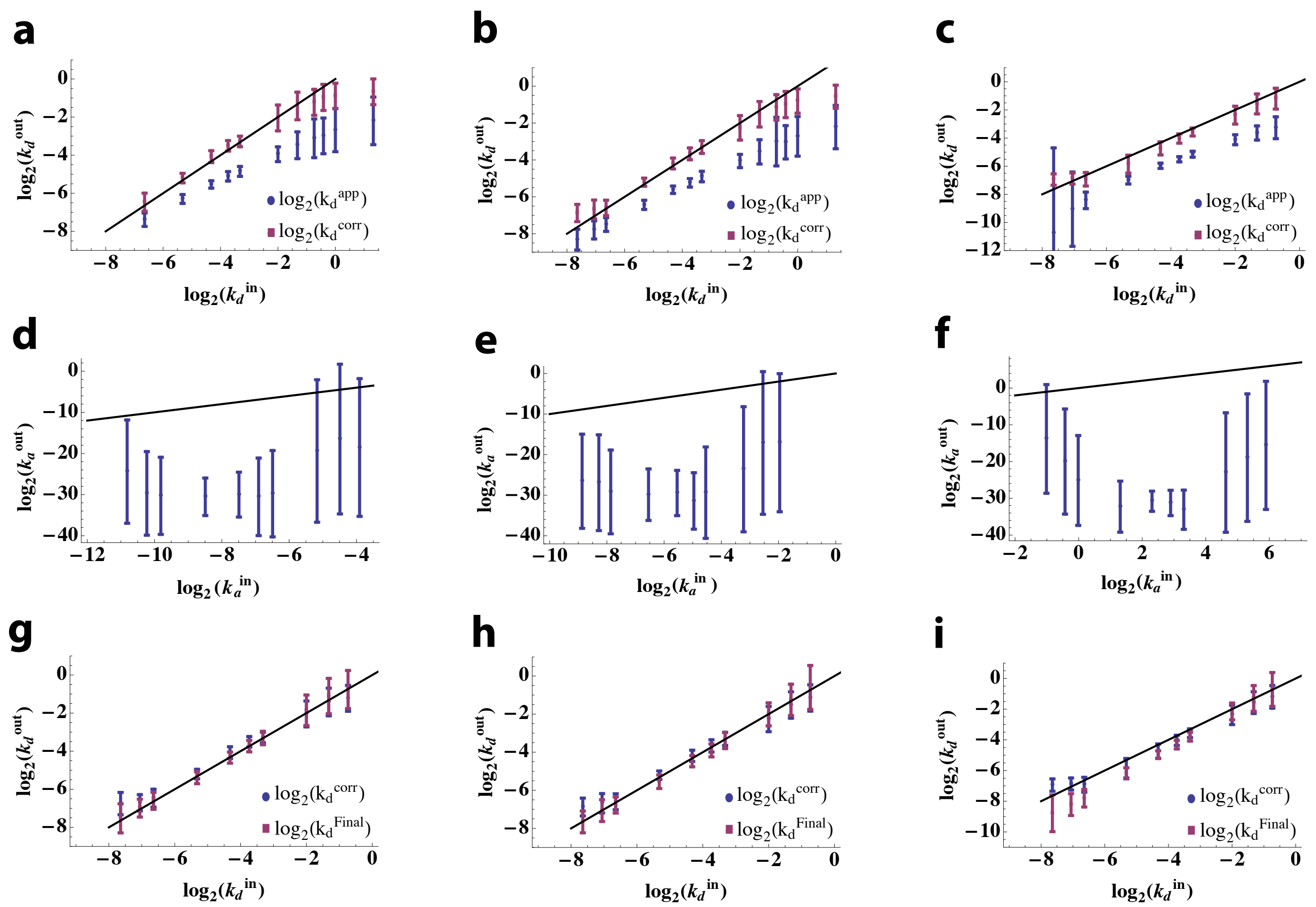

Supplementary Figure S2: k_a and k_d estimates at different points of the simulated fitting procedure. **a − c,** $\log_2(k_d^{app})$ and $\log_2(k_d^{corr})$ against $\log_2(k_d^{in})$ for various values of θ_b. **a,** $\theta_b = 0.1$, **b,** $\theta_b = 0.3$, **c,** $\theta_b = 0.99$. The black line shows the reference ideal case with no error $k_d^{out} = k_d^{in}$. k_d^{app} is the k_d extracted from fitting the ideal competition ChIP solution that assumed instantaneous induction of induced protein to the simulated data. k_d^{corr} is the corrected value of k_d extracted from the look-up table generated by estimating the bias in k_d^{app} over a wide range of k_d values. The figures show that the ideal solution systematically underestimated k_d, while k_d^{corr} was an excellent approximation to the input k_d, emphasizing that the look-up table provides a good estimate for the next part of our fitting algorithm: one-dimensional Newton's method fit of the differential equations to the competition ChIP data. **d − f,** $\log_2(k_a^{out})$ against $\log_2(k_a^{in})$ for various values of θ_b. **d,** $\theta_b = 0.1$, **e,** $\theta_b = 0.3$, **f,** $\theta_b = 0.99$. The black line shows the reference $k_a^{out} = k_a^{in}$. k_a^{out} is order of magnitude off from k_a^{in} for a wide range of θ_b values, once again showing that is it not feasible to extract k_a from ratio (R_m^n) data. Since the concentration of the endogenous TBP was not an independently measured quantity, we absorbed the concentration of endogenous TBP in the definition of k_a giving k_a units of $1/min$ (as we have done throughout our analysis). **g − i,** Simulated $\log_2(k_d^{corr})$ and $\log_2(k_d^{final})$ against $\log_2(k_d^{in})$ for various values of θ_b. **g,** $\theta_b = 0.1$. **h,** $\theta_b = 0.3$. **i,** $\theta_b = 0.99$. The black line shows the reference $k_d^{out} = k_d^{in}$. k_d^{corr} is the value of k_d estimated from the look-up table, while k_d^{final} is the value of k_d returned from one-dimensional Newton's method fitting starting from k_d^{corr} as an initial estimate. The figures show that we reproduced k_d^{in} reliably as k_d^{final} over a wide range of θ_b. In the simulations and the look up table the data was simulated 200 times for each value of input parameters assuming normal noise with a standard deviation of 0.2.

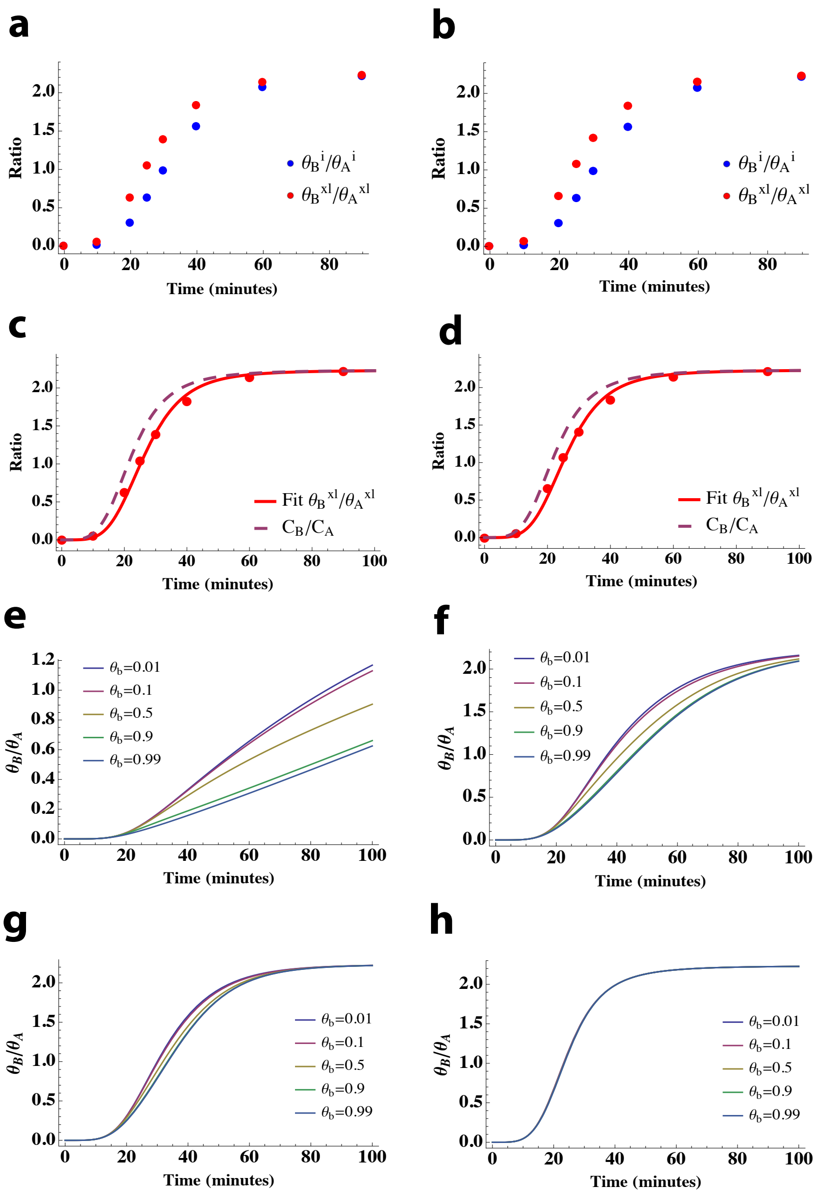

Supplementary Figure S3: **a − d,** Comparison of θ_B^i/θ_A^i (in vivo ratio of occupancies of competitor to endogenous TBP) and $\theta_B^{xl}/\theta_A^{xl}$ (cross-linked ratio of occupancies of competitor to endogenous TBP). The effect of cross-linking for twenty minutes was numerically simulated using Eqs. (4) - (8). **a − b,** Comparison of the crosslinked ratio to the in vivo ratio with $k_d = 0.1$/min, $\theta_b = 0.1$, **a,** $k_{xl} = 0.1$/mol min, **b,** $k_{xl} = 10$/mol min. **c − d,** Fits of the in vivo differential equations to the crosslinked ratio with $k_d^{in} = 0.1$/min, $\theta_b = 0.1$, **c,** $k_{xl} = 0.1$/mol min gives $k_d^{out} = 0.26$/min, **d,** $k_{xl} = 10$/mol min gives $k_d^{out} = 0.28$/min, which shows that ignoring cross-linking could overestimate k_d by about a factor of 3. **e − h,** Simulation of θ_B^i/θ_A^i for various values of k_d and $\theta_A^i(t \to 0)$ ($\theta_A^i(t \to 0) = k_a/(k_a + k_d)$, where we have absorbed the concentration of endogenous TBP into k_a, similar to what we have done throughout the analysis). Equivalently, for a given k_d, $\theta_A^i(t \to 0)$ determined k_a. **e,** $k_d = 0.01$/min, **f,** $k_d = 0.05$/min, **g,** $k_d = 0.1$/min, **h,** $k_d = 0.5$/min. The occupancy ratio (and by extension, R_m^n) was almost completely independent of k_a (i.e., θ_b) for $k_d \geq 0.05$/min. Only the ratio for slow k_d (panels **e** and **f**) may give reliable values for k_a (in the ideal case of no noise in the data), which constituted less than 8% of the total loci we identified. Hence, we did not extract k_a in this study.

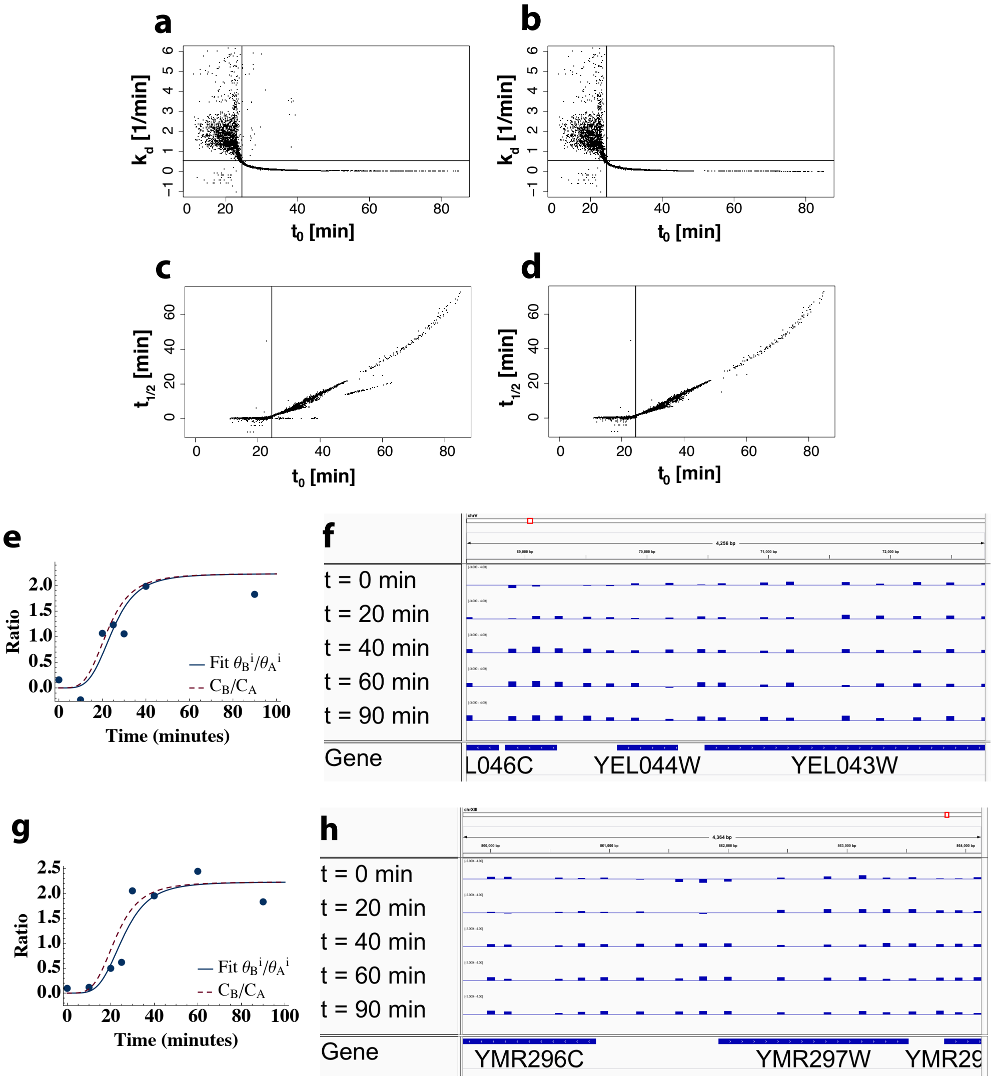

Supplementary Figure S4: k_d and $t_{1/2}$ before and after fixing fitting exceptions, and sample fits to the data. **a,** k_d against t_0 before corrections as described in Supplementary Text Sec. 5, **b,** k_d against t_0 after corrections, **c,** $t_{1/2}$ against t_0 before corrections, and **d,** $t_{1/2}$ against t_0 after corrections. The vertical bar is the reference $t_0 = 24.5$ minute. Reliable estimates of k_d and $t_{1/2}$ were less than 0.5/min and greater than 83 seconds, respectively. **a** and **c** show that fitting exceptions and failures occurred at a small fraction of the total number of sites analyzed. **b** and **d** show that our modifications to the algorithm starting point and/or the step size fixed the failures and produced consistent, reliable values of k_d. **d** also highlights that t_0 can be crudely approximated as $t_0 \simeq t_0^{ind} + t_{1/2}$: the figure shows a nearly linear, monotonic relationship between t_0 and $t_{1/2}$ with slope near 1 for $t_0 \gtrsim 25$ min. **e** $-$ **h,** Fits to the data along with IGV plots of $\log_2(R_m)$ (before normalization and locus-specific background subtraction and scaling). **e, f,** YEL043W with $t_{1/2} = 1.4$ min. **g, h,** YMR297W with $t_{1/2} = 2.2$ min. The IGV plots show $\log_2(R_m)$ going from negative to positive as induction time increases signifying an increase of the competitor relative to the endogenous TBP at a binding locus.

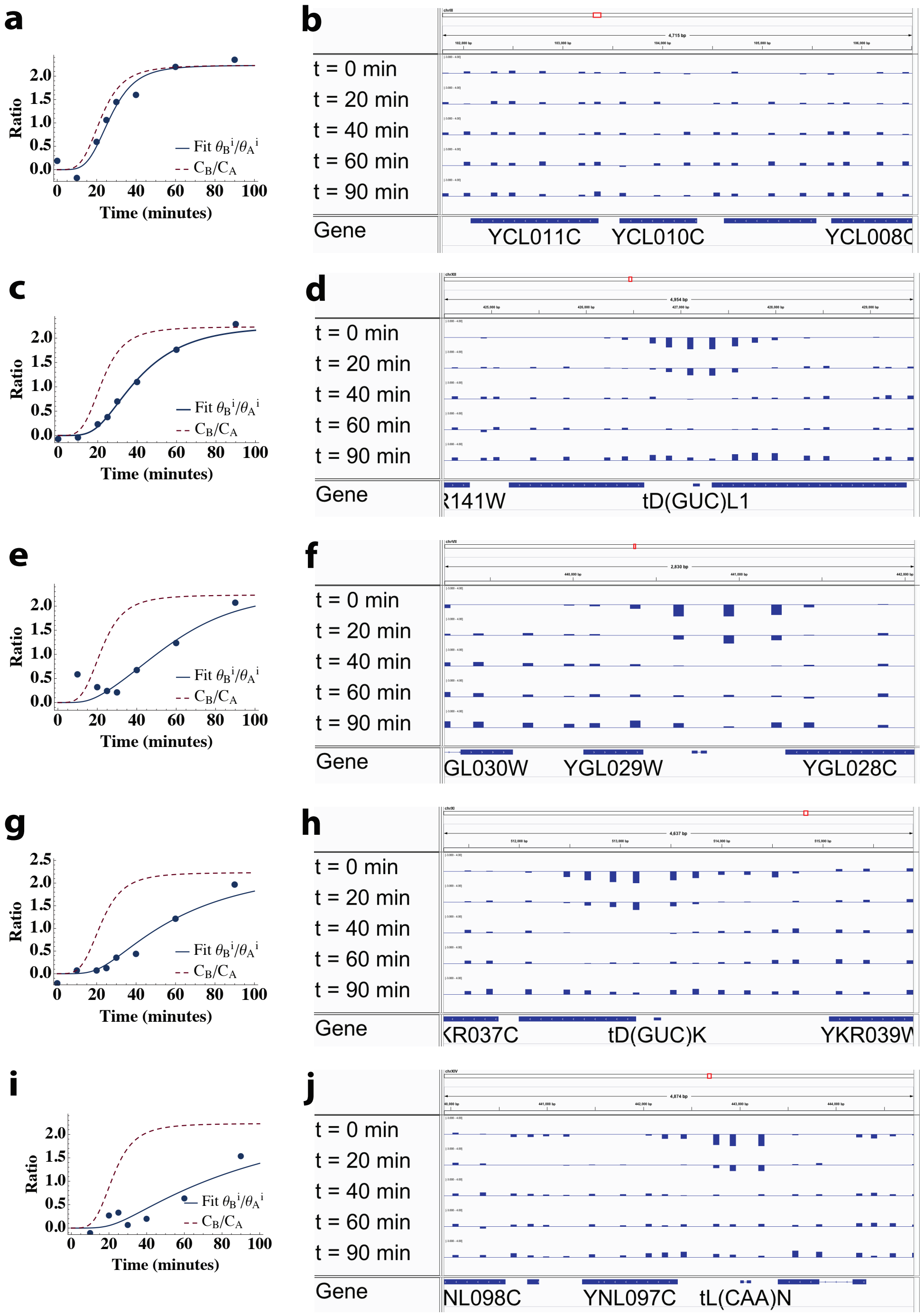

Supplementary Figure S5: Continuation of Supplementary Figure S4 showing fits to the data along with IGV plots of $\log_2(R_m)$. **a, b,** YCL010C with $t_{1/2} = 3.0$ min. **c, d,** tD(GUC)L1 (top strand) with $t_{1/2} = 13.9$ min. **e, f,** YGL029W with $t_{1/2} = 18.6$ min. **g, h,** tD(GUC)K (bottom strand) with $t_{1/2} = 29.9$ min. **i, j,** YNL097C with $t_{1/2} = 53.3$ min.

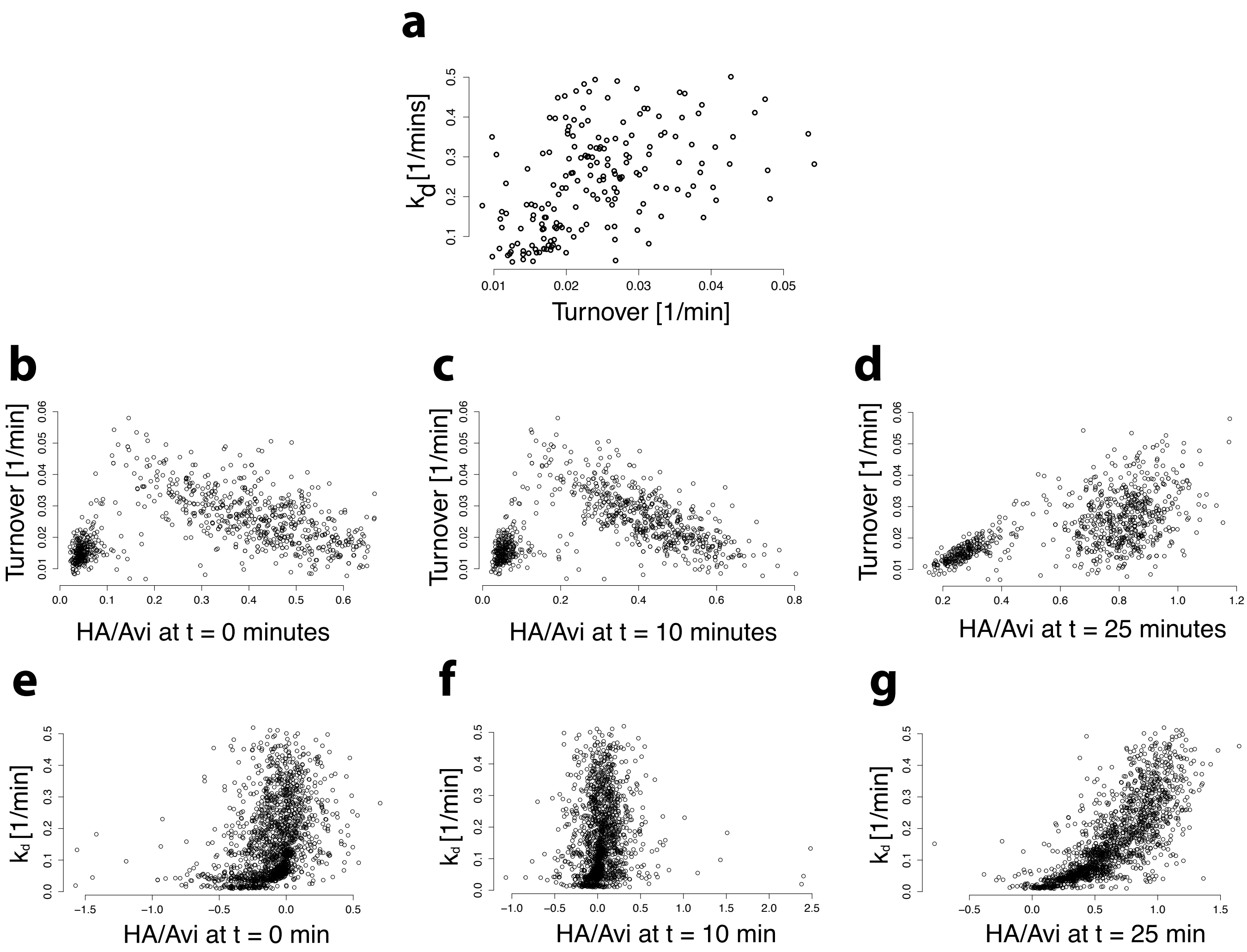

Supplementary Figure S6: Comparison of fitting procedure in this study with that of van Werven et al. [1]. **a,** Correlation between TBP k_d and turnover parameter (r) from van Werven et al. [1]. The figure shows that k_d and r were correlated, as one would expect, but the scales were different by a factor of 10. That highlights two things: one, that our normalization and scaling procedures were different from van Werven et al. [1], and more importantly, that a careful consideration of protein induction (as in our kinetic model) was necessary to extract physical residence times. $\mathbf{b-d}$**,** Dependence of turnover (r) from van Werven et al. [1] on S_{HA}/S_{Avi} for induction times of 0 minute, 10 minutes, and 25 minutes. $\mathbf{e-g}$**,** Dependence of k_d on HA/Avi signal for induction times of 0 minute, 10 minutes, and 25 minutes. HA/Avi signal was the background subtracted, normalized and scaled signal (R_m^n) that we used in all our analyses. Pol III genes tended to be in the lower left corner of $\mathbf{b-d}$ with both low turnover and HA/Avi values, and Pol II genes were the remaining points in these scatter plots. The correlation between r and HA/Avi for Pol II genes in van Werven et al. [1] goes from negative to positive as we go from induction time of 0 minute to 25 minutes. This suggests that the data in Ref.[1] may not be properly normalized. k_d reported in this study was not correlated with the data at 0 minute and 10 minutes induction times because of background subtraction.

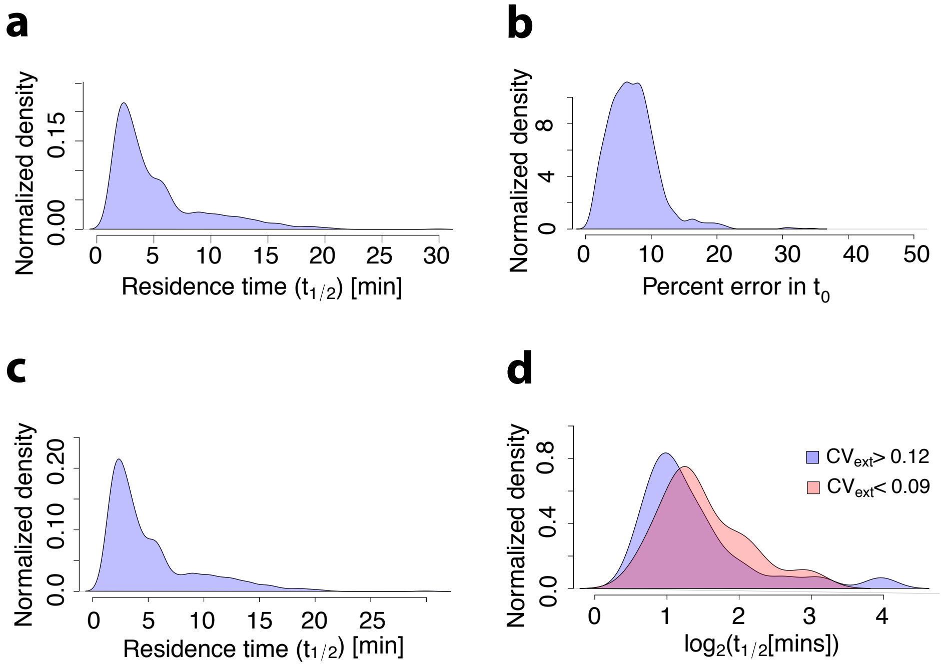

Supplementary Figure S7: Genome-wide distribution of residence times ($t_{1/2}$), distribution of the percent error in response time (t_0), and the effect of removing the loci with the most imprecise t_0 (and $t_{1/2}$) estimates on downstream analyses. **a,** The distribution of $t_{1/2}$ among the 999 Pol II and Pol III loci that are reported in Supplementary Table S1. **b,** The percent error in the response time (100× standard error in t_0 divided by t_0) is of the order of a few percent, with a median of 6.9%. The standard error for a locus (Δt_0) was estimated from a sigmoid fit to the normalized, scaled ratio data using linearized approximations around the best fit as implemented in the NonlinearModelFit function of Mathematica. **c,** To see how the error in the fits affected the genome-wide residence times, we re-plotted the histogram of $t_{1/2}$ excluding all loci for which the percent error in t_0 was greater than 15% (39 loci). The genome-wide distribution of $t_{1/2}$ is similar to that in **a**. **d,** We re-analyzed the result presented in Fig. 4e with the reduced dataset of the loci in **c**. The result of Fig. 4e is robust to this test, with a new p-value of 0.04 compared to the original p-value of 0.048. Hence, the biological results presented in the paper are robust to our least precise t_0 (and $t_{1/2}$) values.

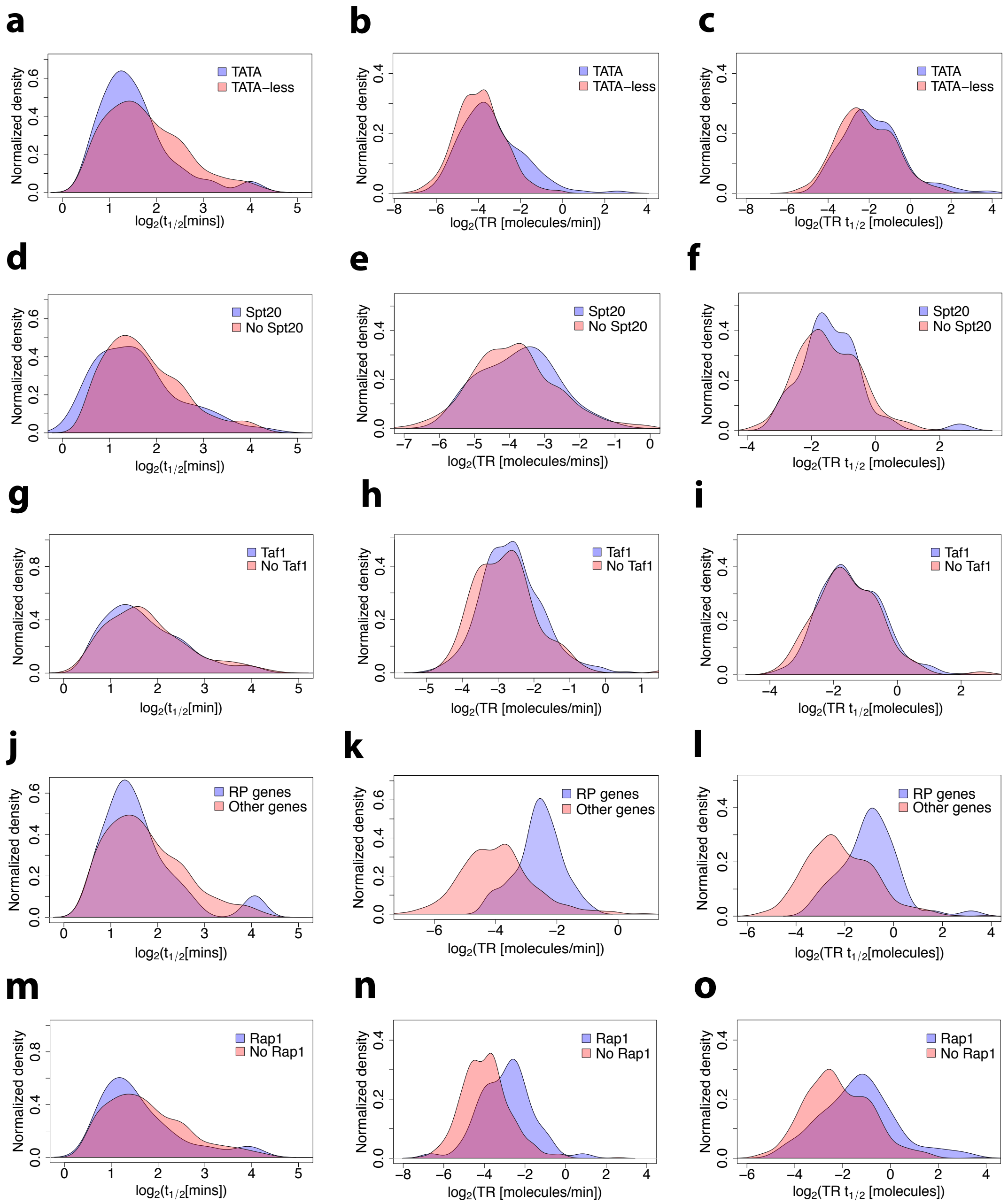

Supplementary Figure S8: Residence times ($t_{1/2}$), transcription rate (TR), and transcription efficiency (TR $t_{1/2}$) for **a – c,** TATA containing versus TATA-less, **d – f,** SAGA-bound versus SAGA-less, **g – i,** TFIID-bound versus TFIID-less, **j – l,** RP versus non-RP, and **m – o,** Rap1 bound versus other loci. As expected, RP genes had a very high transcription rate compared to other Pol II genes. In fact, RP genes transcribe nearly 1 RNA molecule per TBP residence, which is ~ 5 fold more efficient than other Pol II genes. Presence of Rap1 did not affect TBP residence times, but did increase the transcription rate and the transcription efficiency.

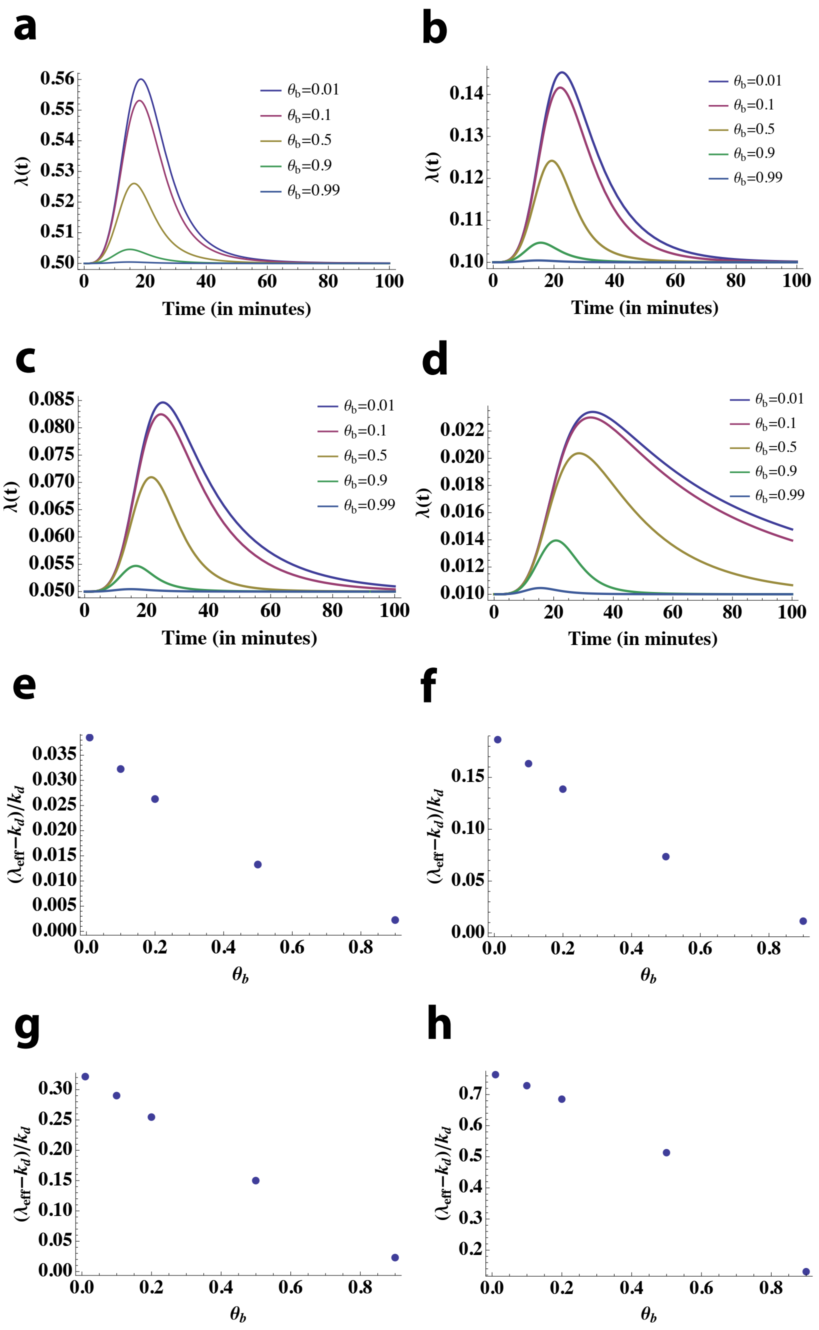

Supplementary Figure S9: **a − d,** $\lambda(t)$ for k_d between 0.5/minute and 0.01/minute, which covered the range of k_d values we found from competition ChIP data [1]. The x-axis is the induction time. **a,** $k_d = 0.5$/min. **b,** $k_d = 0.1$/min. **c,** $k_d = 0.05$/min. **d,** $k_d = 0.01$/min. Lickwar et al. [4] assume that λ is a constant, but the figures show that this assumption is true for occupancies approaching 1. For expected occupancies in the range of $\theta_b \sim 0.1$ to $\theta_b \sim 0.5$, we see that λ is a function of the induction time. **e − h,** The error induced in k_d by assuming that $\lambda(t)$ is a constant and equals k_d, for k_d between 0.5/minute and 0.01/minute. **e,** $k_d = 0.5$/min. **f,** $k_d = 0.1$ / min. **g,** $k_d = 0.05$/min. **h,** $k_d = 0.01$/min. For each of the occupancies $\theta_b = 0.01, 0.1, 0.5, 0.9, 0.99$, we calculated λ_{eff} as the average of λ over the induction time points of 0, 10, 20, 25, 30, 40, 60, and 90 minutes. λ_{eff} is an estimate of the λ returned by the Poisson-model fit to the competition ChIP data. The figure shows that for small k_d (high residence times), the assumption could induce an error of up to a factor of 2 in k_d estimates.

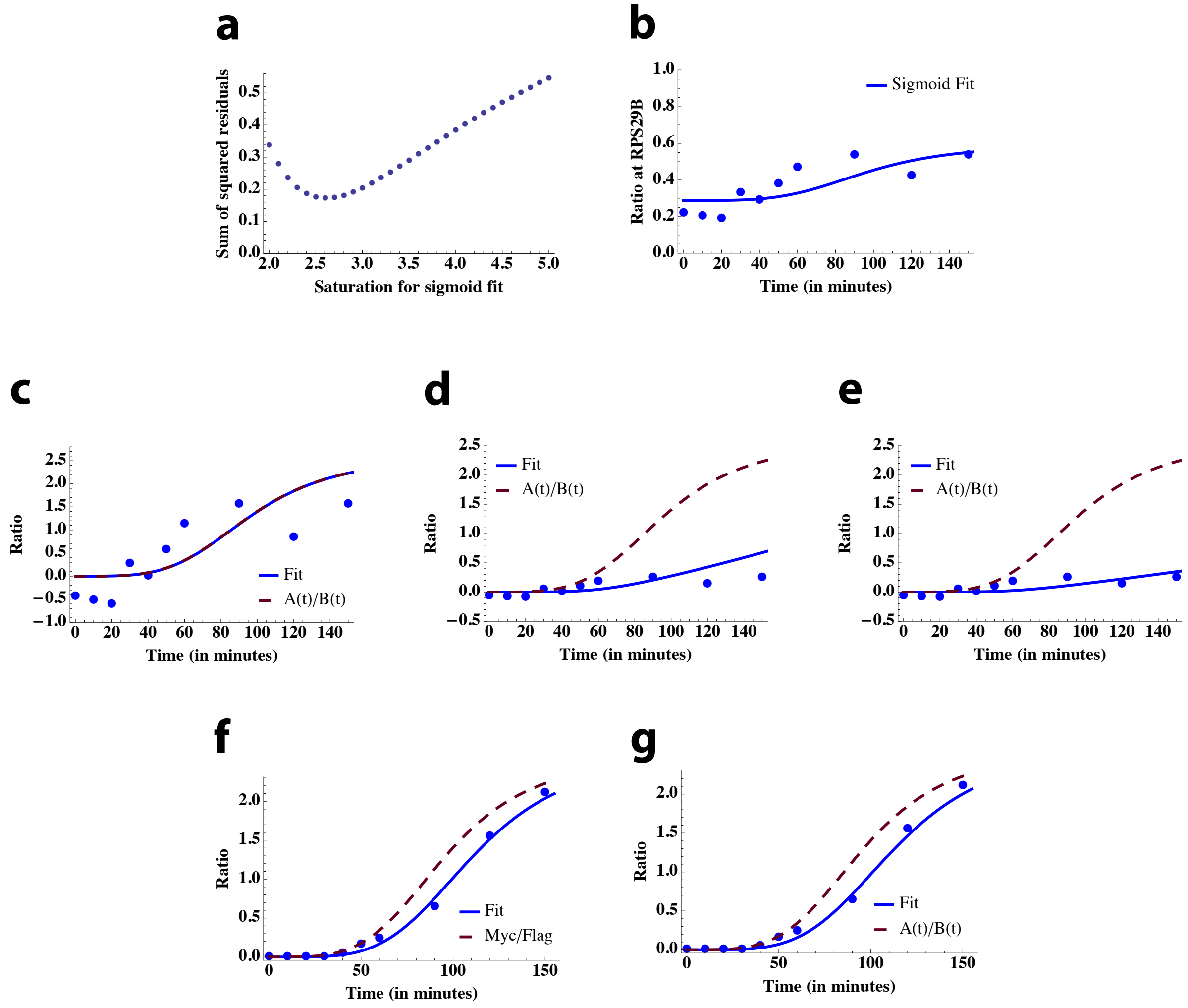

Supplementary Figure S10: Comparison of fitting procedure with Lickwar et al. [4]. **a** − **b,** Sigmoid fits to Myc/Flag concentration ratio and to RPS29B data in Lickwar et al. [4]. **a,** The minimum of the residuals of sigmoid fit to Myc/Flag ratio was at a saturation value of ~ 2.6. **b,** Sigmoid fit to RPS29B data highlighted that the data saturated at ~ 0.6. **c** − **e,** Fits of the RPS29B data from Lickwar et al. [4] using our kinetic model. **c,** Fit with data scaled. **d,** Numerical curve with input $1/k_d$ of 99 min. **e,** Fit to unscaled data resulting in $1/k_d = 200$ min. **d,** and **e,** show that $1/k_d$ of 200 min that we got fitting the kinetic model was comparable with $1/\lambda$ of 99 min that Lickwar et al. [4] find fitting the Poisson turnover model to RPS29B Rap1 data. **f** − **g,** Fits of kinetic model to simulated Rap1 data from Fig. S6.f in Lickwar et al. [4]. **f,** Our kinetic fit predicted $1/k_d$ of 882 seconds. **g,** Curve with $1/k_d$ of 1000 seconds overlaid on the simulated Rap1 data to show that the curve was almost indistinguishable from the curve with $1/k_d = 882$ seconds. Hence, our kinetic fit value of 882 seconds was within the noise error to the input λ^{-1} of 1000 seconds to the simulated Rap1 data.

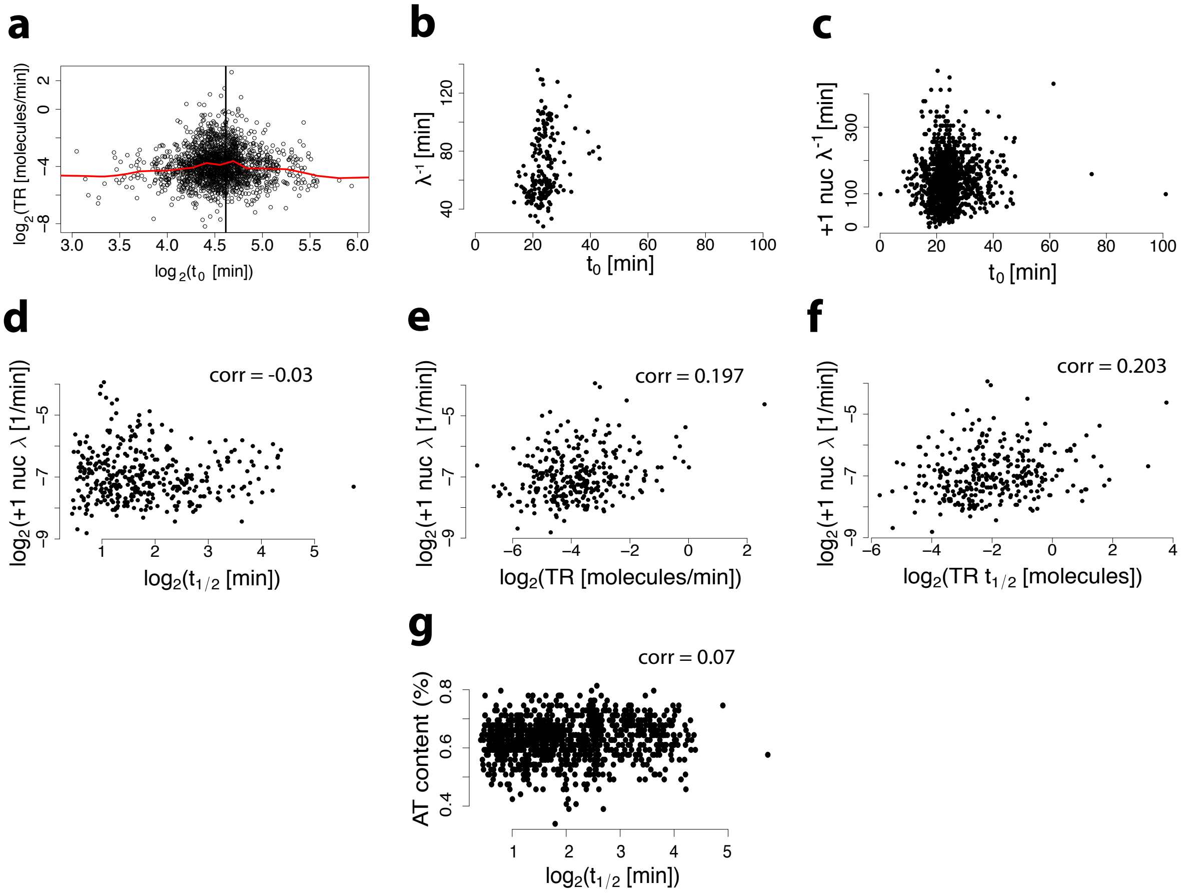

Supplementary Figure S11: Transcription rate, Rap1 and +1 nucleosome relative residence time, and AT content (%) against t_0 (or $t_{1/2}$) for competition ChIP loci. t_0 is used as a proxy for $t_{1/2}$. **a,** Transcription rate versus t_0, where the red curve is a LOESS smoothed average of the transcription rate. A non-monotonic relationship between t_0 and transcription rate is shown, which would also imply a non-linear relationship between $t_{1/2}$ and transcription rate. **b,** Rap1 residence time versus t_0. The figure shows that most of the Rap1 sites were concentrated near the fast edge of our data set, which will have to be confirmed with more comprehensive data sets that quantitatively explore loci with TBP residence times of a minute or less. **c,** +1 nucleosome λ^{-1} from Dion et al. [7] versus t_0 of TBP competition ChIP loci. **d − f,** +1 nucleosome turnover against $t_{1/2}$, transcription rate, and transcription efficiency. +1 nucleosome turnover was mildly correlated with the transcription rate (**e**) and transcription efficiency (**f**). **g,** The AT content of the probe sequence is not correlated with TBP residence time.

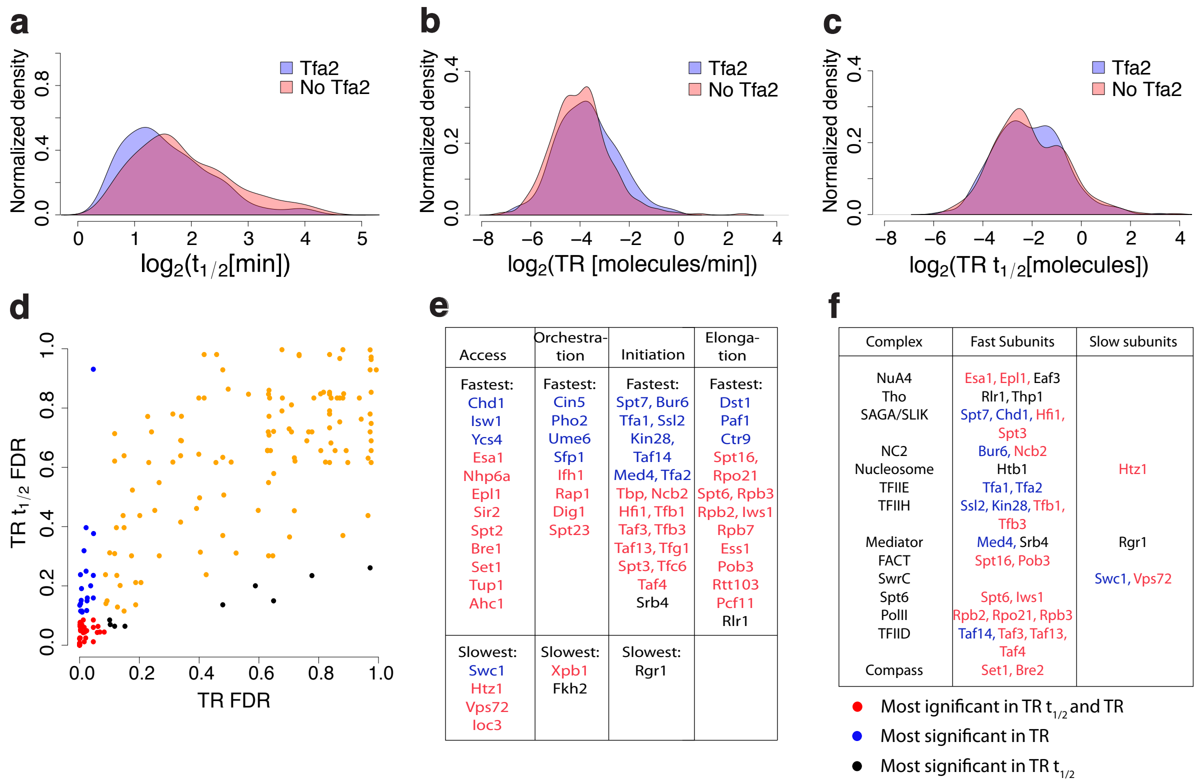

Supplementary Figure S12: Occupancy of many TFs modulates transcription rate and efficiency, but not TBP residence time. **a–c,** The presence of Tfa2 yielded **(a)** lower TBP residence time $t_{1/2}$ (KS p-value=$5.4e-3$) and **(b)** transcription rate (TR) with KS p-value=0.028, while **(c)** the transcription efficiency $TRt_{1/2}$ was not affected by Tfa2. Tfa2 was the only TF whose presence affected TBP residence time out of 202 mapped TFs. **d,** Significance of the genome-wide change in TR (x-axis) and TR$t_{1/2}$ (y-axis) due to the presence or absence of each TF (each dot) of 202 mapped TFs [5]. The false discovery rate (FDR) was calculated by performing a permutation test between the loci where a TF was bound and the loci that lacked that TF, after which multiple hypothesis correction (Benjameni-Hochberg correction) was applied. Low FDRs were used to identify TFs that are likely to affect the transcription rate only (blue dots), the transcription efficiency only (black dots) or both (red dots). **e,** Ranked list of TFs plotted in **(d)** (categorized according to access, orchestration, initiation and elongation) with the highest (Fastest) or lowest (Slowest) TR (blue), TR$t_{1/2}$ (black), or both TR and TR$t_{1/2}$ (red). **f,** Select multi-protein complexes from the list in **(e)** highlighting the role of each complex in increasing (Fast Subunits) or decreasing (Slow Subunits) TR and/or TR$t_{1/2}$.

References

[1] F. van Werven et al., Nat. Str. and Mol. Bio. **16**, 1043-1048 (2009)

[2] V. Pelechano, S. Chavez, and J. Perez-Ortin, PLOS one, 0015442 (2014)

[3] Wolfram Research Inc., Mathematica version 8.0, Champaign, Illinois (2010).

[4] C. Lickwar et al., Nat. **484**, 251-255 (2012).

[5] B. Venters et al., Mol. Cell **41**, 480-492 (2011)

[6] K. Poorey et al., Science, **342**, 369-372 (2013)

[7] M. Dion et al., Science, **315**, 1405-1408 (2007)

[8] A. Nakao, M. Yoshihama, and N. Kenmochi, Nucleic Acids Res, **32**, 168-170 (2004), *http://ribosome.med.miyazaki-u.ac.jp/*

[9] O. J. Rando et al., Genetics **190**, 351-387 (2012)

[10] M. S. Santisteban et al., Mol. Cell. Biol., **31**, 1848-1860 (2011)
